# Inclusion Complexes Between α-Cyclodextrins and Xenon: Molecular Dynamics Simulations and Experimental Study

**DOI:** 10.3390/molecules31193554

**Published:** 2026-10-06

**Authors:** Francesco Trotta, Eya Ben Khalifa, Fabrizio Caldera, Adrián Matencio, Gjylije Hoti, Federico Cesano, Giuseppina Raffaini

**Affiliations:** 1 Department of Chemistry, University of Torino, Via P. Giuria 7, 10125 Torino, Italy; francesco.trotta@unito.it (F.T.); eya.benkhalifa@unito.it (E.B.K.); fabrizio.caldera@unito.it (F.C.); adrian.matencio@um.es (A.M.); gjylije.hoti@unito.it (G.H.); 2 Department of Chemistry, Materials and Chemical Engineering “Giulio Natta”, Politecnico di Milano, Piazza Leonardo da Vinci 32, 20133 Milano, Italy

**Keywords:** xenon, α-cyclodextrin, inclusion complexes, molecular dynamics simulations, xenon release

## Abstract

Xenon (Xe) is an inert noble gas receiving increasing attention in medical research due to its anesthetic properties, ability to regulate metabolic processes, and broad organoprotective effects. Due to its small size and non-covalent intermolecular interactions, Xe can be encapsulated within α-cyclodextrin (α-CD). In this study, we combine a theoretical study based on molecular mechanics (MM) and molecular dynamics (MD) simulations with the synthesis and characterization of the α-CD/Xe inclusion complex. During the MD simulations, surface interactions and the formation of inclusion complexes, with Xe atoms encapsulated within the α-CD cavity, were observed in aqueous solution. As the Xe concentration in water increased, α-CD/Xe inclusion complexes with 1:1, 1:2, and 1:3 stoichiometries were formed and remained stable over time, suggesting increasingly effective encapsulation at higher pressures. Following this computational investigation, experimental work was conducted to synthesize and characterize the α-CD/Xe inclusion complex using a liquid-phase encapsulation method. The complexes were prepared at Xe loading pressures of 2, 4, 6, and 8 bar. Thermogravimetric analysis (TGA) revealed weight loss between 100 and 200 °C. The effective encapsulation of Xe was also confirmed by solid-phase microextraction coupled with gas chromatography–mass spectrometry (SPME-GC-MS), which detected the characteristic m/z signals of Xe and allowed monitoring of its release over time upon contact with aqueous solution.

## 1. Introduction

Xenon (Xe), a noble inert gas present in trace amounts in the atmosphere [1], is distinguished by its rapid pharmacokinetics, low blood/gas partition coefficient and non-toxicity [2]. This medical gas combines anesthetic, neuroprotective, and cardioprotective properties, acting through competitive inhibition of glutamatergic N-methyl-D-aspartate (NMDA) receptor at glycine binding sites [3,4]. Recent studies prove the therapeutic potential of Xe in Alzheimer’s disease and tauopathy [5,6]. Xe has also shown promising results for treating Parkinson’s disease due to its neuroprotective and anti-dyskinetic properties [7]. Despite its promising therapeutic profile, xenon delivery faces two challenges: first, its high cost and poor aqueous solubility; second, its inertness due to a filled valence shell, which excludes covalent bonding. This inertness is counterbalanced by high polarizability (4.04 Å^3^), enabling strong London dispersion forces and van der Waals interactions [8]. These non-covalent interactions constitute the primary driving forces for Xe’s inclusion within suitable host molecules, enabling stable clathrate formation. Xe binding studies include multiple host molecules: hemicarcerands [9], cryptophanes for biosensing applications [10,11,12,13], porous organic cages for rare gases separation [14], metallic organic frameworks for gas storage and Xe/Kr separation [15,16], and chiral capsules for enantioselective recognition [8]. Although diverse Xe host candidates exist, these synthetic materials suffer from poor aqueous solubility, multi-step synthesis procedure, and toxicity concerns that limit their biomedical applications. Cyclodextrins (CDs) overcome these limitations as FDA and EMA approved materials with high aqueous solubility and a relatively hydrophobic cavity that selectively accommodates diverse guest molecules [17]. α-cyclodextrin (α-CD) consists of six α-1,4-linked glucopyranose units arranged in toroidal geometry [18]. This shape confers a conical form with a hydrophilic outer and a slightly hydrophobic inner cavity [19]. The combination of approved safety profile, high aqueous solubility and well-defined cavity size makes α-CD particularly suitable for hosting different gaseous molecules, such as ethylene [20,21], CO_2_ [22], 1-methylcyclopropene [23], nitrogen, nitrous oxide [24] and krypton [25]. In this context, Xe stands out as an attractive guest because of its high polarizability with a Van der Waals diameter compatible with the dimensions of the α-CD cavity, thus favoring its encapsulation. 

This work presents computational modeling followed by experimental validation of α-CD/Xe inclusion complexes. The theoretical investigation is based on molecular mechanics (MM) and molecular dynamics (MD) methods at the atomistic level using a protocol proposed in previous studies on host–guest complexes involving cyclodextrins [26,27,28,29,30,31]. Intermolecular interactions between α-cyclodextrin and xenon were theoretically investigated at different initial α-CD/Xe ratios to elucidate the possible formation of α-CD/Xe inclusion complexes (ICs) at different stoichiometries during the MD simulations. The intermolecular interactions induce the encapsulation in the small cavity of the α-CD in aqueous solution, which is consistent with experimental findings from NMR experiments reported in the literature [32,33]. Favorable intermolecular interactions occur between the Xe atoms and the outer surface of the α-CD and between the xenon atoms themselves at higher concentrations. The experiment was performed using liquid-phase encapsulation. The effects of pressure and loading time were studied, and the obtained inclusion complex was characterized through thermogravimetric and FTIR analysis. The release of Xe from the complex upon contact with water was monitored using SPME-GC-MS. Theoretical results followed by experimental data are presented in the following section.

## 2. Results and Discussion

### 2.1. Theoretical Study of α-CD/Xe Interactions

Initially, MM/MD simulations considering α-CD interactions with Xe atoms in a random arrangement at different ratios were performed *in vacuo* to understand possible encapsulation in stable α-CD inclusion complexes. Following analysis of the theoretical results, MM/MD simulations in water were performed to better understand the role of water molecules in the encapsulation of xenon atoms in α-CD, particularly at higher Xe concentrations. Water molecules stabilize the inclusion process. The theoretical results are presented and discussed in the next paragraphs.

#### 2.1.1. Theoretical Study of α-CD/Xe Interactions *In Vacuo*

Using a simulation protocol adopted in previous works [26,30,31] and discussed in Section 3, the initial non-optimized geometries considering an α-CD and Xe atom(s) at 1:1, 1:2, 1:3, 1:10 and 1:30 ratios were considered, as shown in Figures S1 and S2. After energy minimization, Xe atom(s) become encapsulated, leading to the formation of inclusion complexes involving α-CD and Xe atom(s) with 1:1, 1:2, and 1:3 stoichiometries, as shown in panels (a), (b), and (c) in Figure 1, respectively—along with the corresponding van der Waals and electrostatic energy contributions listed alongside. The favorable contribution due to the Coulombic term is relevant.

In the α-CD/Xe IC with a 1:1 stoichiometry (Figure 1a), the distance between the Xe atom and the center of mass (c.o.m.) of the α-CD is equal to 0.10 Å. The intramolecular H-bonds calculated for isolated α-CD in vacuo are 19; likewise, there are 19 intramolecular H-bonds in IC with a 1:1 stoichiometry in Figure 1. The inclusion of the Xe atom did not alter the number of intramolecular H-bonds between the atoms of α-CD. Considering the α-CD/Xe IC with a 1:2 stoichiometry (Figure 1b), the distance between the Xe atom closest to α-CD secondary rim is equal to 1.25 Å, while the distance between the Xe atoms closest to the primary rim is 2.68 Å. The distance between the two Xe atoms is 3.93 Å. Considering the α-CD/Xe IC with a 1:3 stoichiometry (Figure 1c), the distance between the Xe atoms well encapsulated within the hydrophobic cavity is 0.17 Å, while the distances from the CD c.o.m. and the other two Xe atoms closest to the secondary edge are 4.26 and 4.58 Å, respectively. Of particular interest is the mutual arrangement of the three Xe atoms, which form an equilateral triangle with sides measuring (4.228 ± 0.252) Å. In the optimized initial geometries of the α-CD/Xe inclusion complexes (ICs) with 1:2, 1:3, and 1:10 stoichiometries, seventeen intramolecular H-bonds are present; this number increases to eighteen for the 1:30 stoichiometry. The initial interaction between α-CD and the Xe atoms, following initial energy optimization, does not significantly alter the structure of the α-CD itself. The non-bond interactions calculated in the optimized geometries are negative due to favorable non-covalent interactions (see the values calculated for the optimized geometries in Figure 1 and Figure 2). A significant contribution is due to Coulombic interactions.

At larger Xe atoms concentrations, after the initial energy minimization, α-CD/Xe IC with a 1:2 stoichiometry is formed, as shown in the encapsulated Xe atoms in green in Figure 2. For the initial α-CD/Xe systems with 1:10 and 1:30 ratios (see panel (a) in Figure 2), the optimized geometries show that the distances between the encapsulated Xe atoms near the secondary and primary rims are equal to 1.13 and 2.72 Å (Figure 2a) and 1.85 and 1.94 Å (Figure 2b), respectively. In both cases studied, the mutual arrangement of the four unencapsulated Xe atoms near the α-CD secondary rim is particularly noteworthy. As shown in dark green in Figure 2, these atoms form an approximate square with side lengths of (4.079 ± 0.085) Å (optimized geometry in panel (a) of Figure 2), and (4.053 ± 0.020) Å (optimized geometry in panel (b) of Figure 2). In the final complex, corresponding to the highest concentration of Xe studied, the formation of an approximately equilateral triangle formed by three Xe atoms adsorbed on the external surface of the cyclodextrin was noteworthy. The triangle had sides equal to (4.182 ± 0.118) Å, similar to that formed by three Xe atoms in the α-CD/Xe IC with a 1:3 stoichiometry, shown in panel (c) of Figure 1. From this analysis of the obtained data, we can therefore highlight the interesting tendency of Xe atoms to aggregate with each other, forming ordered geometric structures in the cavity of the α-CD and on its external surface.

The interaction energy between the α-CD and Xe atom(s) is defined as

*E_int_* = *E*_tot_ − (*E*_α-CD free_ + *E*_Xe atoms free_)

where *E_α-CD free_* and *E*_Xe atoms free_ are the energies of the isolated α-CD molecule and Xe atom(s), respectively, and *E*_tot_ is the potential energy of the optimized α-CD/Xe system *in vacuo*, as shown in Figure 1 and Figure 2. According to this definition, *E_int_* < 0 indicates favorable intermolecular interactions between Xe atom(s) within the CD internal cavity or on its surface exposed to the environment. The magnitude of *E_int_* value represents the energy required to detach the Xe atom(s) far from the α-CD and return it to the free state. In Figure 3, the interaction energy (filled symbols) of the optimized initial geometries—related to the same geometries previously discussed as well as and others involving a single α-CD molecule—and an increasing number of Xe atoms is plotted as a function of the number of Xe atoms in the simulation cell.

*E_int_* decreases linearly with Xe atoms due to favorable non-covalent intermolecular interactions between the internal cavity of the α-CD and the external surface and between the Xe atoms themselves.

Starting from all the initial optimized geometries reported in Figure 1 and Figure 2, the MD runs were performed at a constant temperature (T = 300 K) *in vacuo*. In all cases, the Xe atoms initially encapsulated or adsorbed onto the external surface of the α-CD moved rapidly away from the CD because their kinetic energy exceeded the weak intermolecular interactions. Therefore, these formed inclusion complexes were not stable *in vacuo*. Subsequent calculations in water highlight the role of this polar solvent in promoting the solubilization of cyclodextrin and Xe, particularly at higher concentrations. This suggests that, experimentally, increasing the pressure is advantageous because it increases the amount of Xe in water in contact with α-CD, thereby forming inclusion complexes that are more stable over time than those *in vacuo*. This behavior is in accordance with Henry’s law, which states that at constant temperature, the solubility of a gas in a liquid is directly proportional to the pressure of the gas above the solution.

In the final geometries optimized after the MD runs, the Xe atoms generally adopted a random arrangement in the simulation cell, except for in the α-CD and Xe system at a 1:10 ratio. In fact, as shown in panels (a) and (b) in Figure 4, in this system, three Xe atoms arranged themselves in a nearly equilateral triangle with sides measuring (15.17 ± 0.594) Å around the outer surface of the cyclodextrin. In this geometry, cyclodextrin forms only 7 intramolecular H-bonds, compared to the 19 present in the isolated state or in the previously discussed optimized initial geometries. The van der Waals contributions (specifying the repulsive and dispersive components) and the electrostatic contribution are reported (in kJ/mol) in Figure 4.

#### 2.1.2. Theoretical Study of α-CD/Xe Interactions in Aqueous Solution

Using a simulation protocol proposed in previous works [34,35], the initial geometries considering an α-CD and one or more Xe atoms in ratios at 1:1, 1:2, 1:3, 1:10 and 1:30 were investigated, with the Xe atoms randomly distributed in a cubic cell in the presence of water molecules, as shown in Figures S3 and S4, respectively.

Xe atoms were encapsulated after energy minimization (shown in Figures S5 and S6), molecular dynamics, and optimization of different conformations assumed by the system during MD runs, encapsulation of Xe atoms occurred. We also emphasize that, despite the mobility of Xe at the simulation temperature, the most populated geometries during the molecular dynamics simulation feature the Xe atom included in the α-CD cavity, in particular at high Xe concentration in a simulation cell. The potential energy and Coulomb contribution calculated during MD runs in water starting from the geometry in panels (a), (b), and (c) of Figure S5 and panels (a), and (b) of Figure S6 are reported in panels (d), (e), and (f) of Figure S5 and in panels (c), and (d) of Figure S6, respectively.

Inclusion complexes involving α-CD and Xe atoms with stoichiometries of 1:1, 1:2, and 1:3 formed relatively quickly, as previously found *in vacuo*. In addition, xenon atoms were adsorbed onto the external surface of α-CD and underwent self-aggregation due to favorable intermolecular interactions between the α-CD and Xe atoms, as well as between the xenon atoms themselves, particularly at higher gas concentrations.

For the optimized α-CD/Xe system with a 1:1 stoichiometry (panel (a) in Figure S5), the Xe atom initially included in the hydrophobic cavity of α-CD remained encapsulated throughout most of the molecular dynamics simulation, except in a few conformations sampled during the simulation. Panel (a) of Figure 5 shows the distance between the c.o.m. of α-CD and the initially encapsulated Xe atom calculated as a function of simulation time in ns, starting from the optimized geometry shown in panel (a) of Figure S5. The Xe atom was included throughout almost all molecular dynamics simulations, except for some geometries where the calculated distances were between 4 and 5 Å. When comparing the solvent-accessible surface area (SASA)—the surface is accessible to water molecules—which was calculated for isolated α-CD in water and the 1:1 α-CD/Xe complex (with α-CD in its optimized state at the end of the molecular dynamics simulation in water), a slight decrease in SASA (of approximately 6%) was observed for the α-CD/Xe complex. The SASA values in Å2 are reported in Table S1 of the Supplementary Materials.

Regardless of whether the initial system contains α-CD and two or three Xe atoms (panels (b) and (c) in Figure S5), the inclusion complex forms first with a 1:1 α-CD/Xe stoichiometryand then with a 1:2 α-CD/Xe stoichiometry. Indeed, as reported in panels (c) and (a) of Figures S5 and S7, respectively, the first Xe atom was encapsulated and it remained close to the c.o.m. of α-CD. The second Xe atom was included around 1.2 ns, thus forming the α-CD/Xe complex with a 1:2 stoichiometry. The α-CD/Xe inclusion complexes with a 1:2 stoichiometry formed in water during two different MD simulations, starting at α-CD to Xe ratios of 1:2 and 1:3, as reported in panels (b) and (c) of Figure S7, respectively. After the inclusion of the second Xe atom, the mobility of the first initially included gas atom increased, maintaining a nearly constant distance between the Xe atoms. When comparing the solvent-accessible surface area (SASA) for water molecules—calculated for isolated α-CD in water and different 1:2 stoichiometry α-CD/Xe inclusion complexes (considering the optimized geometries saved during molecular dynamics simulations)—a decrease of approximately from 6% to 11% was observed. The SASA values (in Å^2^) are listed in Table S1 of the Supplementary Materials.

These results could be compared with those of our previous theoretical study on the inclusion of 5-fluorouracil (5-FU), an anticancer drug, into β-CD, initially with a 1:1 stoichiometry and subsequently with a 1:2 stoichiometry in water [34,35]. Interestingly, when a second Xe atom was included in the α-CD cavity, the mobility of the first encapsulated Xe atom increased, even moving away from the α-CD (see Figure 5c and Figure S7a). Consequently, over time, the 1:2 inclusion complex and simple 1:1 α-CD/Xe IC were formed. Notably, in this case study and in the studies on the inclusion of a hydrophobic drug molecule, such as 5-FU, a distinct mobility was observed depending on the non-covalent intermolecular interactions with the cavity of the cyclodextrin or with the cyclodextrin surface exposed to the solvent. Similar behavior was also observed when a second 5-FU molecule was encapsulated over time within the β-CD cavity in water [35].

Figure 5b,d show the radial distribution function (RDF) [26,27,28] calculated during the MD simulations with the formation of α-CD/Xe ICs with 1:1 and 1:2 stoichiometries. The distance of the Xe atoms calculated from the c.o.m. of the α-CD are within the values of 2 Å. Therefore, over time, conformations with Xe atoms encapsulated within the CD cavity became predominant, as also indicated by the previously discussed graphs showing the variation in the distances between Xe and α-CD. This region of space, where there is a higher probability of finding Xe atoms during MD simulations with respect to the c.o.m. of the a-CD, will not be occupied by water molecules on average.

Figure 5e,f show the RDF of Xe atoms as a function of their distance, r (in Å), calculated over several molecular dynamics simulations of α-CDs and Xe atoms with ratios of 1:2 and 1:3, 1:10 and 1:30, respectively. Interestingly, at low and high Xe concentrations, the mutual distances between Xe atoms are comparable if they are encapsulated within the internal cavity of the CD or interact more with the secondary or primary edge of the α-CD, or more generally, with its external surface. Figure S8 shows the side and top views of the optimized geometries sampled during molecular dynamics simulations in water, starting from α-CD and Xe atom ratios of 1:10 and 1:30. These images illustrate the importance of non-covalent interactions within the hydrophobic cavity of the α-CD and on the external surface, including at the secondary and primary rims. Of particular interest are the ordered structures that formed during the MD runs, including the formation of a row of Xe atoms starting from the α-CD cavity and the arrangement of three Xe atoms that formed an equilateral triangle with side lengths of 4.0 Å (see panels (c1) and (c3) in Figure S8). This triangular arrangement is oriented towards the wider, secondary rim of the α-CD, as previously observed in the geometries optimized in vacuo (see all panels in Figure S8).

Interestingly, the radial distribution function between Xe atoms reported in panels (e) and (f) of Figure 5 calculated over different MD runs exhibits a similar behavior when two or three Xe atoms are considered in the simulation cell or a larger number—specifically 10 or 30—is considered. In these cases, we observe the dynamic formation of a statistically populated 1:2 α-CD/Xe inclusion complex, with Xe atoms at a most probable distance of less than 5 Å (see the RDF in panel e of Figure 5). Conversely, when 10 or 30 Xe atoms are considered in the simulation cell, interactions between Xe atoms occur not only within the cyclodextrin cavity but also on its external surface; consequently, the RDF displays two maxima—one at distances below 5 Å and another in the 6–9 Å range. This second maximum corresponds to distances between Xe atoms adsorbed on the α-CD surface, which interact with one another via weak intermolecular forces. These adsorbed Xe atoms reduce the water-accessible surface area—as shown in Table S1 and represented in Figure S9—resulting in a 16% to 21% decrease in SASA when 6 to 9 Xe atoms interact with the α-CD cavity and external surface.

From all the cases studied, we can therefore conclude that, due to non-covalent interactions, stable geometries in water favor interaction with both the internal cavity and external surface of the α-CD, thereby reducing the surface area accessible to water as the interaction proceeds.

At low xenon concentrations, the oxygen atoms of water molecules can be observed to approach the internal cavity of the CD (see the peak at 1.75 Å and the second peak at 2.73 Å in panel (a) of Figure 6). At higher Xe concentrations, however, access to the internal cavity from the primary and secondary rims of the α-CD becomes sterically hindered by the inclusion, adsorption and self-aggregation process between the Xe atoms themselves due to favorable non-covalent intermolecular interactions, as discussed above (see panels (a2), (b2), (c2), (e2), and (e3) in Figure S8, and the representation of SASA in Figure S9).

To better understand the hydration of Xe atoms at higher concentrations, it is necessary to consider the spatial distribution around the α-CD center of mass (c.o.m.). At higher Xe atom concentrations, the complexes formed during MD simulation comprise encapsulated Xe atoms interacting with each other and with the α-CD atoms in the inner cavity, as well as Xe atoms adsorbed on the α-CD’s outer surface that aggregate with one another; consequently, the entire complex becomes hydrated at greater distances from the c.o.m. Specifically, for α-CD to Xe ratios of 1:10 and 1:30, we compared the RDF of water oxygen atoms relative to the α-CD c.o.m. (see panel (b) in Figure 6) with the RDF of Xe atoms relative to the α-CD c.o.m. (see panel (a) of Figure S10) and calculated the RDF of all α-CD atoms from their c.o.m. (see panel (b) of Figure S10). Indeed, moving outward from the α-CD center, the encapsulated Xe atoms, the α-CD atoms, and the adsorbed Xe atoms form a complex system subject to hydration by water. Interestingly, at these Xe concentrations—with either 10 or 30 atoms—the hydration of the only Xe atom in water is very similar (see panel (c) of Figure S10). These RDFs are similar to those calculated during MD simulation involving a single hydrated Xe atom in a cubic cell containing 1200 water molecules, but in the absence of α-CD. An image of a hydrated Xe atom in the first hydration shell of water molecules is shown in panel (c) of Figure S10, where H-bonds are shown as green sticks. Panel (d) reveals that, on average, water molecules approach Xe atoms at short ranges, primarily through their hydrogen atoms. The hydration of a single Xe atom is analogous, regardless of whether it is considered a single atom or at higher concentrations, where it forms a complex with α-CD via intermolecular forces. It is interesting to note that the novelty is the hydration of Xe in complexes α-CD when we consider a small number of Xe atoms; in fact, the hydration of Xe is substantially similar to that of α-CD (see the three RDFs, which practically overlap in panel (a) of Figure 6) when the α-CD/Xe inclusion complex forms during MD simulation. A plausible explanation is that at low concentrations of Xe atoms, the complexes that can formed with Xe preferentially inside the α-CD cavity are consequently hydrated by the same water molecules that normally hydrate the cyclodextrin (forming a first more ordered hydration shell, a second more extended shell, and a third more disordered shell), effectively behaving as a single unit together with the α-CD—one complex. The void space in the SASA calculated for different α-CD/Xe ICs at a low Xe concentration reported in Figure S10 could visually explain how the encapsulated Xe atoms could be consequently hydrated by α-CD water molecules around the cyclodextrin itself (see Figure S11) [36].

### 2.2. Experimental Validation of α-CD/Xe Inclusion Complex

Panels (a) and (b) in Figure 7 present the TGA and DTG curves of α-CD and the inclusion complex α-CD/Xe obtained at 30 and 60 min, respectively. A first weight loss below 100 °C is observed for all three materials, attributed to water molecules dehydration [37]. The weight loss related to this step was almost the same (Table 1), indicating that Xe inclusion has only a minor impact on the amount of weakly bound water to α-CD. However, a second distinct weight loss appears only in the inclusion complexes α-CD/Xe curves, between 100 °C and 180 °C. This region is quite high in temperature for additional physiosorbed water molecules dehydration and is also well below the temperature of CD degradation. Therefore, this weight loss is likely related to the Xe release from the cyclodextrins cavities. Jessen et al. (2023) have mentioned that less strongly bound water was released at lower temperature and the encapsulated methane in cyclodextrin is more firmly bound than water [38]. The percentage of weight loss in this temperature range is almost identical for the complexes prepared with 30 and 60 min of xenon exposure (Table 1). The encapsulation time did not change the Xe encapsulated within the α-CD cavities, but a longer loading time (60 min) led to a larger quantity of crystal complex being produced. Similar behavior was mentioned by Ho et al. (2011) for ethylene encapsulation in α-CD, where increasing pressure and exposure time did not enhance the ethylene content of the complexes, but mainly increased the overall amount of crystalline inclusion phase obtained [20]. Finally, a decomposition step of the cyclodextrin framework was observed around 280–400 °C, with almost identical onset temperatures and DTG peak shapes for all the samples [39]. This confirms that xenon inclusion in α-CD does not affect the intrinsic thermal stability or degradation pathway of the cyclodextrin.

To evaluate the effect of Xe loading pressure on the thermal stability of the host, TGA/DTG analyses were carried out on α-CD and α-CD/Xe complexes prepared at 2, 4, 6 and 8 bar (panels (a) and (b) in Figure 8). The TGA/DTG analysis reveals a distinct three-step decomposition profile for all Xe inclusion complexes. While α-CD exhibits only two significant weight-loss, dehydration below 100 °C and skeletal decomposition between 250 and 450 °C. All α-CD/Xe complexes display an additional intermediate weight-loss step between 100 and 200 °C, absent in the pristine α-CD. This additional thermal degradation is attributed to the release of water molecules reorganized within the cyclodextrin cavity because of Xe encapsulation, providing indirect thermogravimetric evidence for successful Xe inclusion. The relative weight loss associated with this intermediate step varies with the Xe loading pressure (Table 2), increasing up to 6 bar before slightly declining at 8 bar, suggesting a progressive saturation of the host cavity with increasing gas pressure. Ho et al. (2015) confirmed that higher pressure facilitated the inclusion of ethylene gas into CD molecules, due to increased gas density and penetration of gas to the CD cavities that were not easily accessible under lower pressure [40].

ATR spectra of the α-CD and of the α-CD/Xe sample prepared at a Xe loading pressure of 4 bar, are reported in Figure 9. The two spectra display essentially the same bands. Since Xe is IR inactive, has a small molecular size and is present at relatively low loading, its inclusion may be only able to cause slight vibrational change in the cyclodextrin bands rather than new absorptions bands [20,41]. In the fingerprint region, characteristic bands of α-CD were observed in both spectra, including C-H/O-H bending modes (1410 cm^−1^), C-O-C stretching of glycosidic bond (1151 cm^−1^), C-O stretching (1020 cm^−1^) and skeletal vibration involving α-1,4 linkage (949 cm^–1^) [42,43,44,45]. The signal appearing around 2920 cm^−1^ in both spectra, at the same position and relative intensity, is attributed to the C–H stretching vibrations (symmetric and asymmetric) of the CH and CH_2_ groups of the glucopyranose units. The broad O-H stretching between 3000 and 3600 cm^−1^ in the inclusion complex shows minor changes in shape and shifting to lower wavenumbers compared to the uncomplexed cyclodextrin. This behavior can be related to the reorganization of the hydrogen bond network due to the rearrangement of cavity and lattice water molecules during Xe encapsulation [41,46].

Figure 10a shows the time-dependent increase in Xe peak area during the release of α-CD at four different Xe pressures (2, 4, 6, and 8 bar), performed at proximate physiological conditions. The area of Xe peak was used to quantify Xe release. The narrow and symmetric peak shape, with short retention time (1.15 min), confirms good chromatographic separation and selectivity of the SIM method for Xe detection (Figure 10b). At all pressures, the release curves display the same trend: a rapid initial increase during the first 30 min, followed by a progressive slowing and a plateau at longer times, consistent with a controlled release of Xe. Figure 10 indicates the progressive release of xenon from α-CD/Xe samples over the 90 min following contact with water. This progressive release of encapsulated Xe in aqueous medium indicates that the encapsulation is controlled by weak and non-covalent interactions [47]. This behavior is consistent with recent work on α-CD/Xe solid carriers, where the complex remains stable in the dry state for months but rapidly releases Xe upon contact with water [48]. The α-CD/Xe complex prepared at 2 bar exhibits the lowest peak area, consistent with its comparatively lower weight loss in the intermediate step (4.46%) among all Xe-loaded complexes, both indicating a reduced amount of encapsulated Xe at this pressure. Higher Xe pressure accelerates both the initial uptake rate and the final loading capacity, since a greater partial pressure of gas increases the thermodynamic driving force for guest inclusion into the host cavity, thereby explaining the higher release for pressures 4, 6 and 8 bar. Above 4 bar, however, Xe loading reaches a plateau, likely due to the finite inclusion capacity of the α-CD cavity and steric hindrance caused by Xe adsorption and self-aggregation around the cyclodextrin rims. 

## 3. Materials and Methods

### 3.1. Molecular Mechanics and Molecular Dynamics Simulations

The theoretical study, based on MM and MD simulations at the atomistic level, adopted the same simulation protocol proposed in previous works [31,34,35] for investigating the formation of possible inclusion complexes including CDs and small molecules. First, we considered α-CD and Xe atoms at an initial 1:1 ratio in a cubic simulation cell (side length of 40 Å). Then, systems with stoichiometries of 1:2, 1:3, 1:10, and 1:30 were studied in a random arrangement around the cyclodextrin to assess the role of Xe atom concentration in the non-covalent interactions. Subsequently, additional geometries were examined *in vacuo* by increasing the number of xenon atoms one at a time, thereby obtaining information on the interaction energy of the optimized systems for α-CD/Xe ratios ranging from 1:1 to 1:30. Note that, throughout the manuscript, the ratio between the α-CD molecule and Xe atoms is expressed by indicating the number of α-CD molecules first, followed by the number of Xe atoms, α-CD:Xe. The encapsulation process in the inner hydrophobic cavity and surface interactions with the external part of α-CD occur with one or more Xe atoms. When discussing inclusion complexes, we will always denote the complex as α-CD/Xe in a specific ratio indicated by the first number—which in this manuscript is always 1—followed by the number representing the encapsulated guest atoms.

The simulation protocol consisted of three steps: (I) energy minimization of the initial geometries, with α-CD and Xe in a random arrangement in a simulation box; (II) MD runs at constant temperature (T = 300 K) until an equilibrium state was achieved, and (III) geometry optimization of the conformation assumed by the system at the end of the MD run when an equilibrium state was achieved and selected conformations sampled during the MD runs [26,27,28]. All calculations were performed using the Materials Studio and Discovery Studio packages [49] and the COMPASS force field [50], first *in vacuo* [34] and subsequently in water considering 1200 water molecules in a cubic cell with a side length of 33.5 Å [35]. All energy minimizations were carried out using the Conjugate Gradient algorithm up to an energy gradient lower than 4 × 10^−3^ kJ mol^−1^ Å^−1^. Possible stable α-CD/Xe host–guest complexes in different stoichiometries formed during the MD runs were investigated. After the initial energy minimization, MD runs of 2 ns were performed, followed by final geometry optimizations, allowing all stable and metastable inclusion complexes to be characterized. MD runs lasting 5 ns in vacuo were performed for systems containing ten or thirty Xenon atoms in the simulation cell. The MD simulations were performed in an NVT ensemble (canonical ensemble, see Abbreviations) at a constant temperature (300 K), maintained using a Berendsen thermostat under periodic boundary conditions (PBCs). The integration of the dynamical equations was carried out with the Verlet algorithm using a time step of 1 fs. The conformations assumed by the system were periodically saved and analyzed. The conformations at the end of the MD run when an equilibrium state was achieved, along with other significant conformations assumed by the system, were analyzed and discussed in the main text.

In general, in this work, the initial 1:1, 1:2, and 1:3 ratios of α-CD to Xe atoms are associated with relatively low Xe atom concentrations, whereas the 1:10 and 1:30 ratios occurred at higher Xe atom concentrations; we surmise that, experimentally, this corresponds to the point where Xe atoms at the surface of the incompressible liquid pass into solution due to the increase in pressure.

### 3.2. Materials

α-CD was purchased from CycloLab (Budapest, Hungary). Xe cylinder was supplied by Merck Chemicals GmbH (Darmstadt, Germany) (Purity > 99.99%). Distilled water was used to prepare all the solutions.

### 3.3. Preparation of α-CD/Xe Inclusion Complex

The α CD was lyophilized overnight to remove residual moisture. An amount of 1.4 g of α CD was dissolved in 8 mL of distilled water at 60 °C in the pressure cell. After dissolution, the solution was cooled to 25 ± 1 °C. The Xe cylinder was opened, and the chosen gas pressure was maintained throughout the loading period, under stirring. A white precipitate was formed and collected, frozen in liquid nitrogen then lyophilized overnight. Photographs of the α-CD solution exposed to Xe (6 bar, 25 °C) at t = 0, 2, and 10 min, together with the resulting powder after lyophilization, are shown in Figure S12. 

### 3.4. Characterization

A Perkin Elmer Spectrum 100 FTIR Spectrometer (PerkinElmer Inc., Waltham, MA, USA) equipped with a universal ATR (Attenuated Total Reflection) sampling accessory was used for the recording of FTIR spectra. They were collected in the wavenumber range from 650 to 4000 cm^−1^, with a resolution of 4 cm^−1^. Thermogravimetric analyses were performed using a TA Q500 instrument (TA Instruments, New Castle, DE, USA). The initial weight was around 9 mg. Measurements were performed under nitrogen, at a temperature range from 30 to 700 °C, with a heating rate of 10 °C min^−1^. The release of Xe from the α-CD/Xe complex was evaluated under conditions approximating the physiological environment, at neutral pH and 37 ± 1 °C. Briefly, 10 mg of the inclusion complex was dissolved in 1 mL of distilled water (measured pH 6.4) in a sealed glass vial and was placed in a water bath. The released gas was monitored using SPME-GC-MS. The solid phase microextraction holder was Supelco 57330-U and the SPME fiber was composed of DVB/CAR/PDMS. The gas chromatograph system was an Agilent 6890N GC (Agilent Technologies, Santa Clara, CA, USA), equipped with a 5%-phenyl methyl polysiloxane column. The carrier gas was helium (1.0 mL min^−1^), the split ratio was 1/20 of the total flow and the oven program was 50 °C for 4 min. The mass spectrometer coupled to an Agilent GC apparatus was a 5973 Network Mass Selective Detector (Agilent Technologies, Santa Clara, CA, USA). Mass spectra were recorded under electron impact at 70 eV. The interface was kept at 280 °C, ion source at 230 °C, and quadrupole mass analyzer at 150 °C. Mass spectra were collected in single ion monitoring (SIM), selecting the characteristic ions of the Xe. The instrument was controlled with Enhanced Chem Station (ver. 9.00.00.38) software.

## 4. Conclusions

MM/MD simulations showed that the inclusion of Xe in α-CD at ratios of 1:1, 1:2, and 1:3 is favored in aqueous solution. Non-covalent intermolecular interactions also occurred between Xe atoms and the α-CD external surface and between the Xe atoms, particularly if encapsulated and more exposed to the larger CD secondary rim. Non-bond interactions—van der Waals interactions, weak yet important at short range, and Coulomb forces, which play a significant role—stabilize the inclusion process and surface adsorption over time, maintaining a continuous equilibrium between inclusion complexes (ICs) and complexes in general. Another notable finding is the tendency of the Xe atoms to aggregate together, forming ordered geometric structures within the cavity of the α-CD and on its external surface. During MD simulation, the release of Xe is likely slower for encapsulated Xe atoms than for those interacting on the external surface of the CD, particularly at high Xe concentrations; indeed, the latter tended to move away more easily on average during the MD simulations. The experimental study confirmed the successful encapsulation of Xe within (α-CD) cavities using TGA/DTG, ATR-FTIR, and GC-MS analyses. TGA/DTG revealed an additional weight-loss step (100–200 °C) unique to the α-CD/Xe complexes, attributed to release of water molecules whose organization and interaction with the cyclodextrin host are modified upon Xe encapsulation. The magnitude of this intermediate weight loss varied with the Xe loading pressure, providing indirect evidence of pressure-dependent Xe inclusion. ATR spectra showed only minor changes, mainly in the O–H stretching region, confirming that Xe inclusion involves weak, non-covalent interactions rather than chemical modification of the host. GC-MS analysis demonstrated rapid Xe release upon contact with water. 

These results establish α-CD as an effective host for Xe encapsulation, capable of stable solid-state storage and rapid, controlled release upon hydration for promising applications such as gas storage or Xe delivery. This property may be important in the field of biomarkers and is potentially useful for the study of Alzheimer’s disease (AD) [51,52,53,54]. In recent years, notable studies have investigated the potential utility of novel AD biomarkers. Hane et al. underlined how following inhalation of ^129^Xe atoms, at 60 s after holding their breath, AD patients retained significantly higher amounts of ^129^Xe compared with healthy controls [53]. Theoretical studies and experimental data aimed at improving methodologies and technologies for the encapsulation of Xe atoms in cyclodextrins could represent an important path for the initial study of xenon as a biomarker for Alzheimer’s disease. The rationale behind this work is to explore the possibility of facilitating non-hospital administration of xenon through the oral, pulmonary, and even intravenous routes.

## Figures and Tables

**Figure 1:**
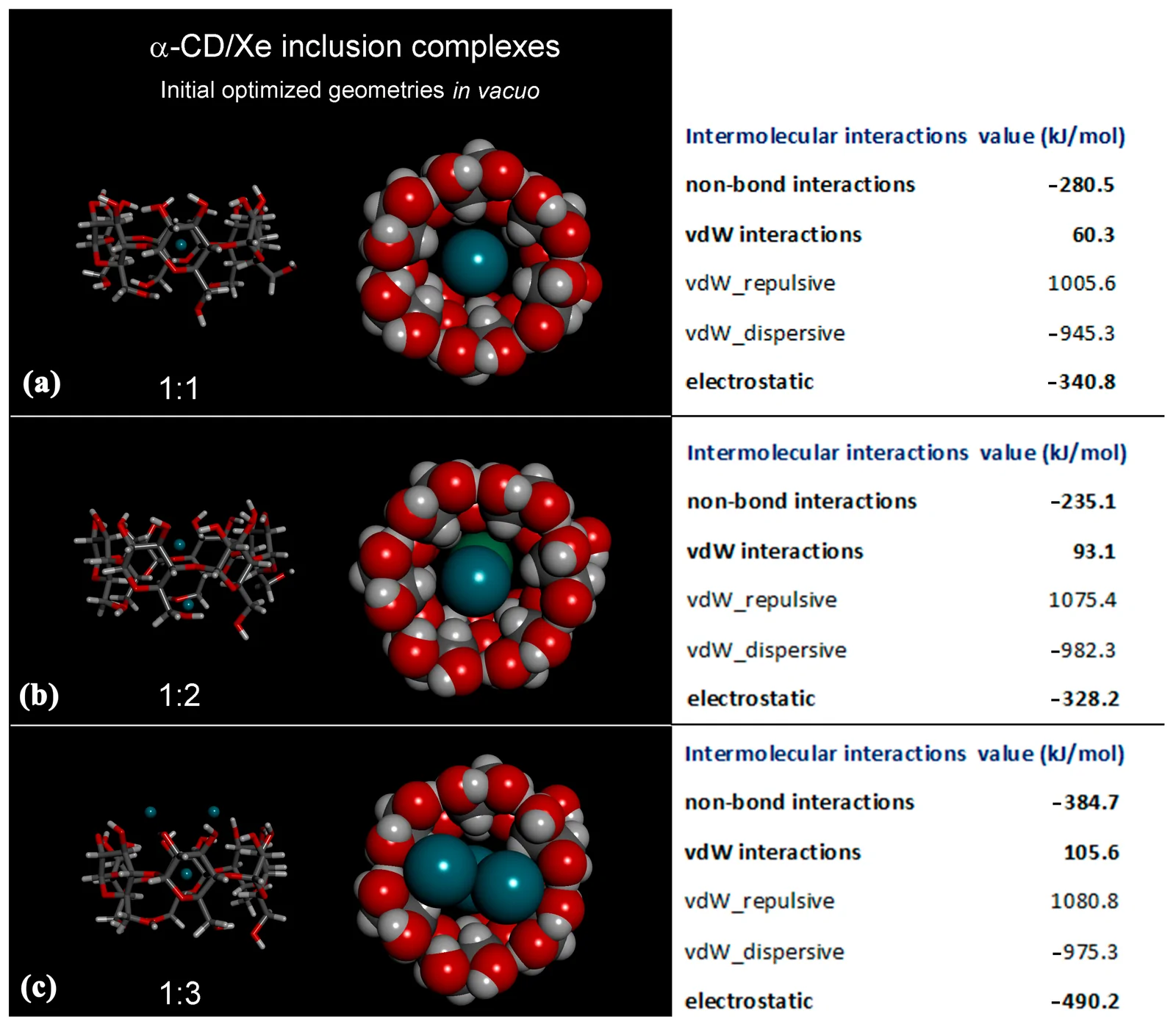
Side and top views, in stick (**left**) and CPK (**right**) representations, respectively, of the initial optimized geometries studied for the interaction between one, two, and three Xe atoms and α-CD in panels (**a**,**b**,**c**), respectively. For the three geometries at the calculated energy minimum, the van der Waals contributions (with its repulsive and dispersive components specified) and the electrostatic contribution are reported (in kJ/mol). Color codes: C atoms are grey, O atoms are red, Xe atoms are petrol blue, and H atoms are white.

**Figure 2:**
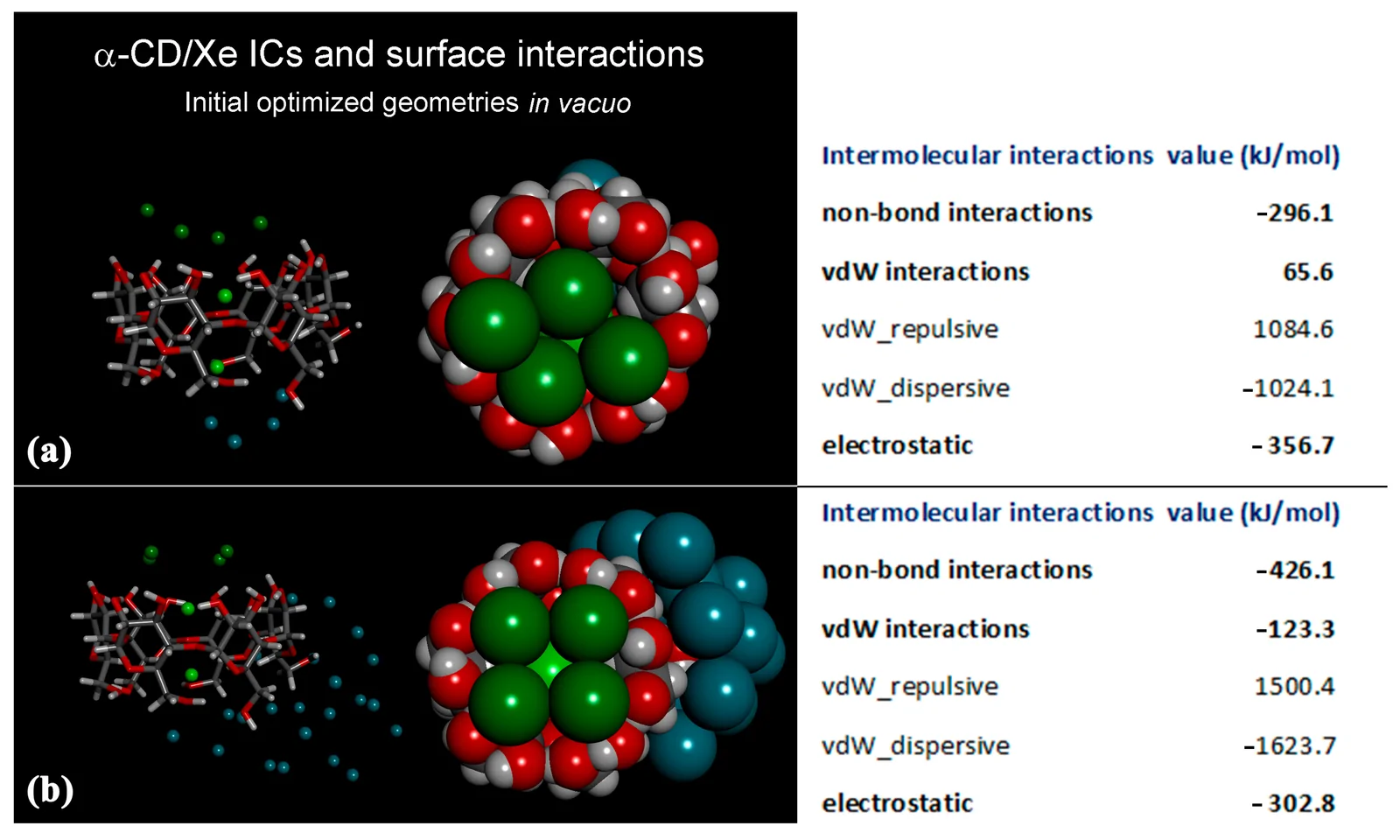
Side and top views, in stick (**left**) and CPK (**right**) representations, respectively, of the initial optimized geometries studied for the interaction between ten and thirty Xe atoms and α-CD in panels (**a**) and (**b**), respectively. For the three geometries at the calculated energy minimum, the van der Waals contributions (with its repulsive and dispersive components specified) and the electrostatic contribution are reported (in kJ/mol). Color codes: C atoms are grey, O atoms are red, Xe atoms encapsulated within α-CD are green, Xe atoms near the α-CD secondary rim are dark green, Xe atoms near the α-CD primary rim and the external surface are petrol blue, and H atoms are white.

**Figure 3:**
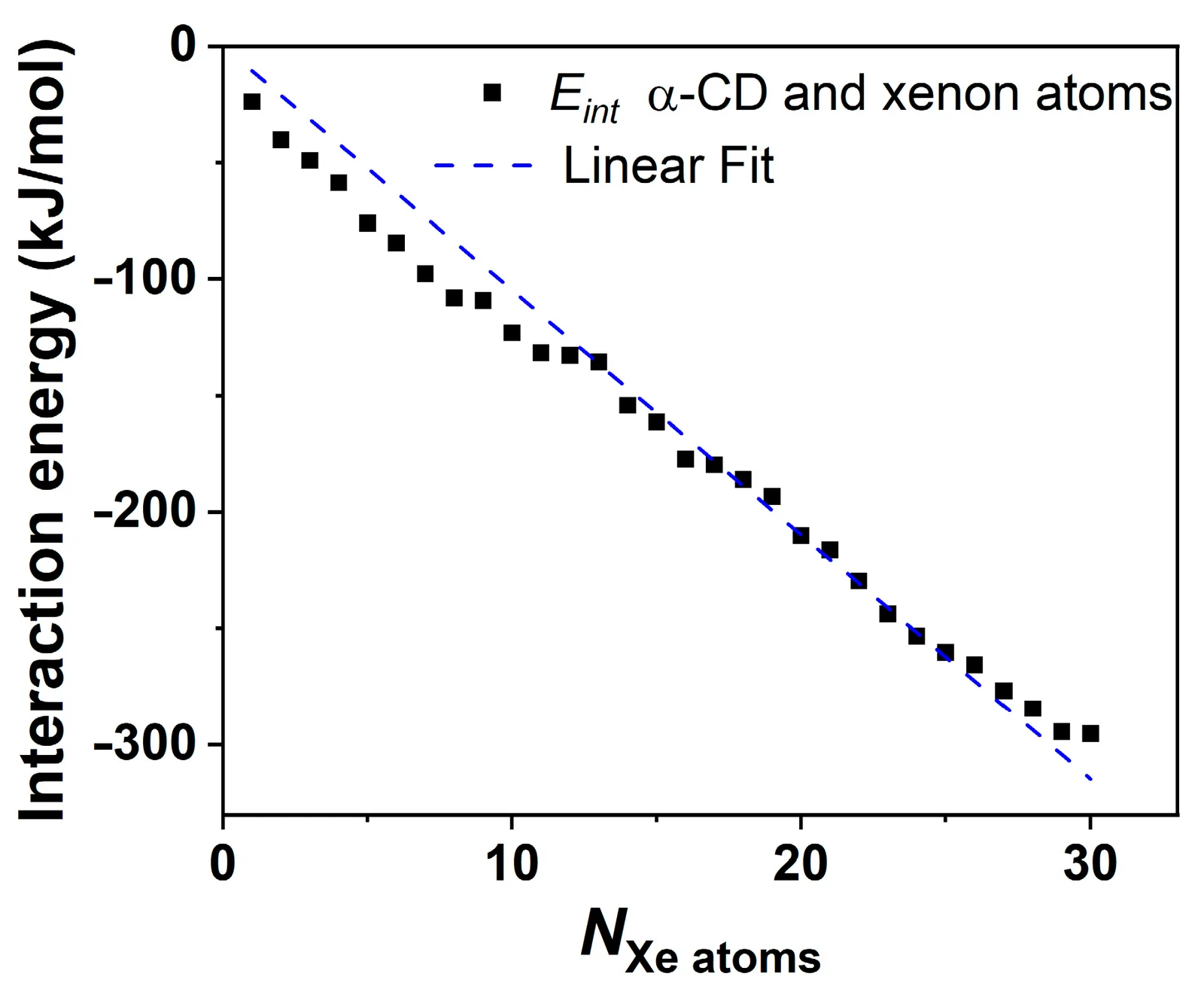
Interaction energy (filled symbols) of the optimized initial geometries reported in Figure 1 and Figure 2, plotted as a function of the number of Xe atoms in the simulation cell. The blue dashed line is the best-fit line through the origin, with a slope equal to (−10.5 ± 0.14) (*R*^2^ = 0.995).

**Figure 4:**
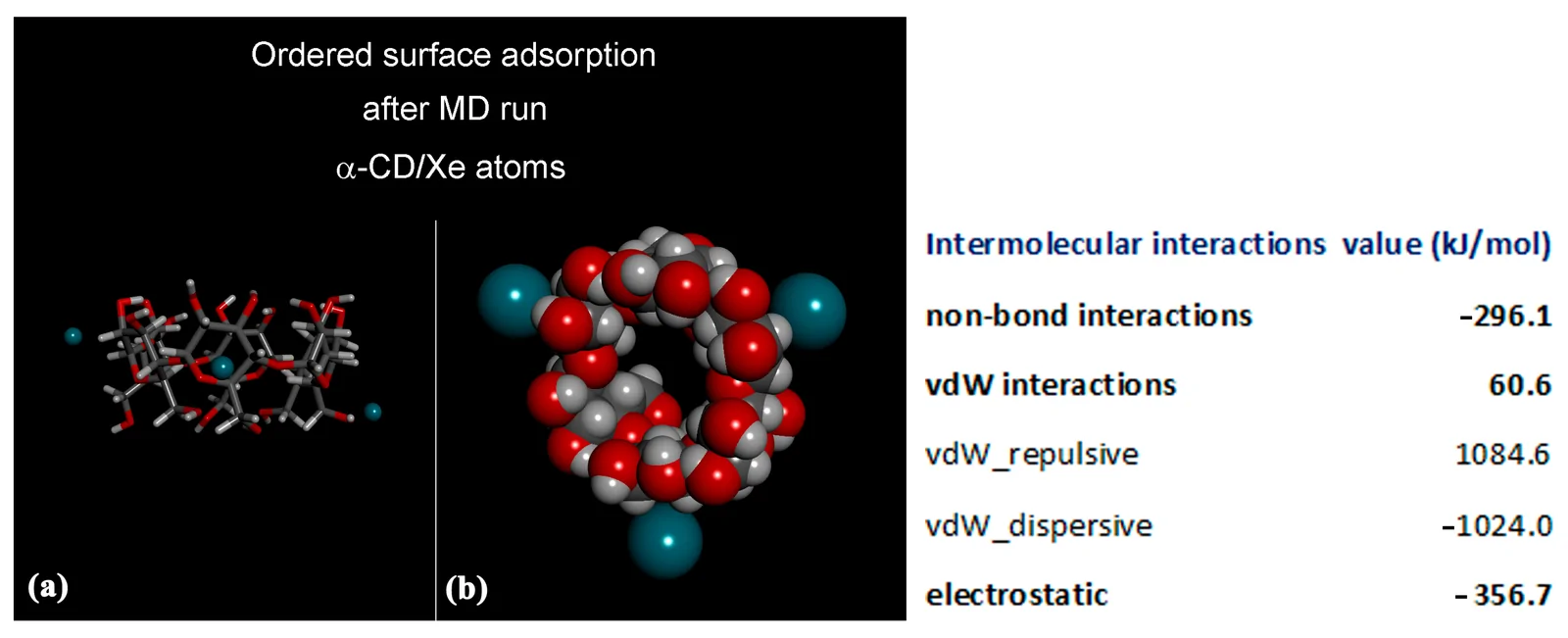
Panels (**a**) and (**b**) show the side and top views, in stick and CPK representations, respectively, of the final optimized geometries after an MD run lasting 5 ns, considering the interaction between ten Xe atoms and α-CD. For this geometry at the calculated energy minimum, the van der Waals contributions (with its repulsive and dispersive components specified) and the electrostatic contribution are reported (in kJ/mol). Color codes are the same as those in Figure 1.

**Figure 5:**
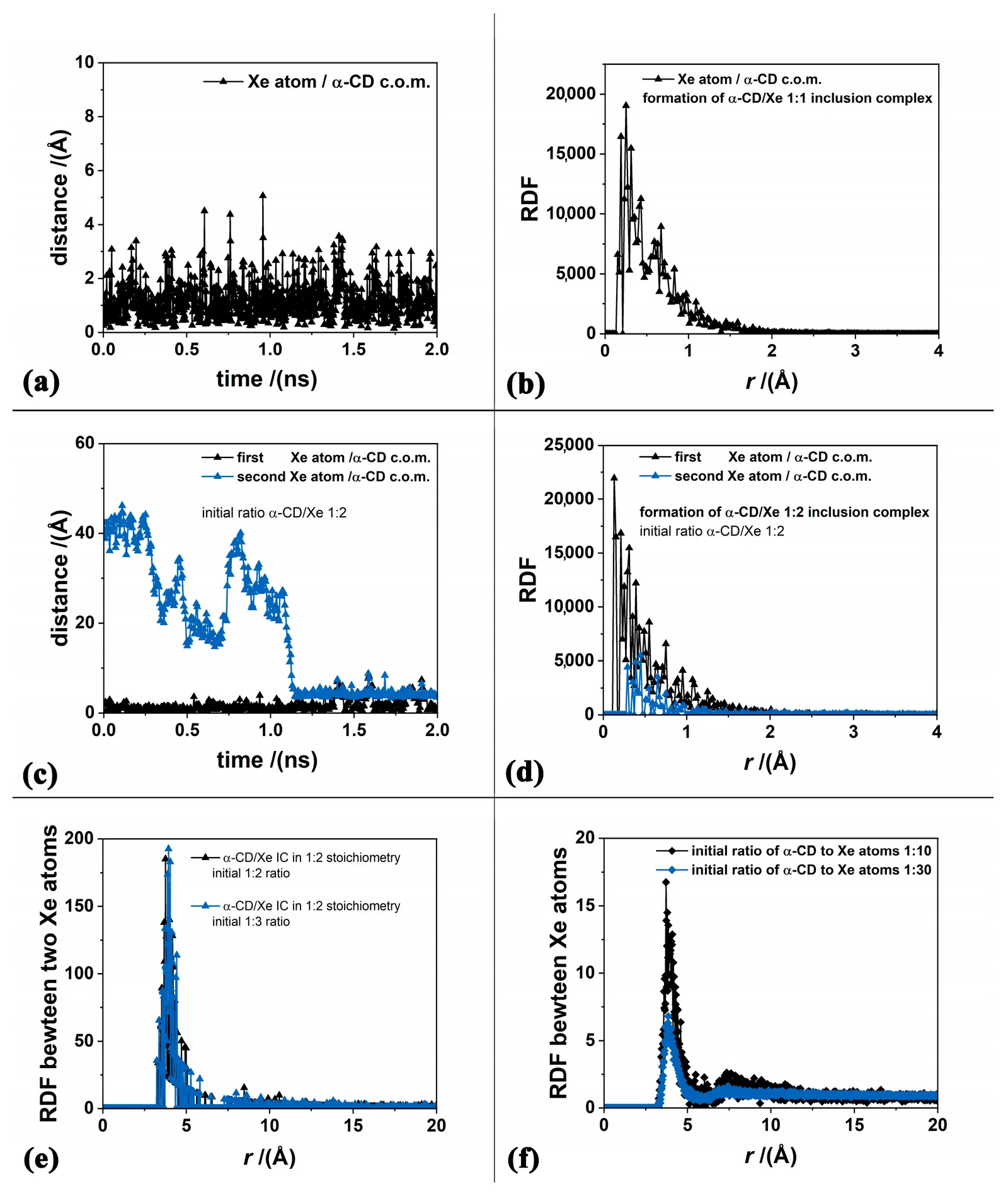
Panels (**a**,**c**): Plots showing the distance of Xe atom(s) from theα-CD c.o.m. as a function of time, calculated during the MD simulations in water considering α-CD/Xe atoms at 1:1 and 1:2 ratios, respectively. Panels (**b**,**d**): Plots showing the RDFs of the oxygen atoms of water molecules as a function of their distance from the α-CD c.o.m., calculated from MD simulations in which ICs with 1:1 and 1:2 stoichiometries were formed, respectively. Panels (**e**,**f**): Plots showing the RDFs of Xe atoms as a function of their distance calculated during MD simulations considering the ratios of 1:2, 1:3 and 1:10, 1:30, respectively.

**Figure 6:**
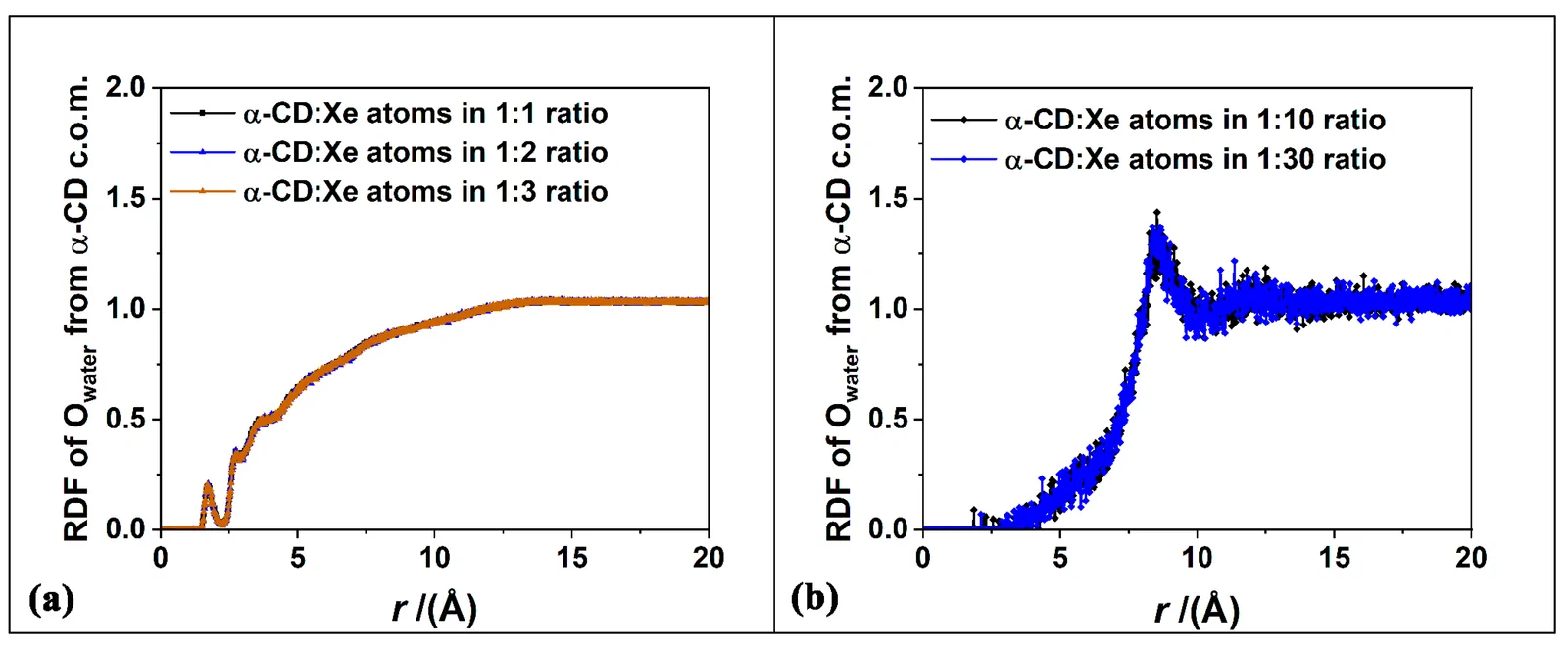
Panels (**a**,**b**): Plots showing the RDF of the oxygen atoms of water molecules as a function of distance, *r* (in Å), from the c.o.m. of the α-CD, calculated during the MD simulation in water for α-CD, with Xe atom ratios of 1:1, 1:2, 1:3 and 1:10, 1:30, respectively.

**Figure 7:**
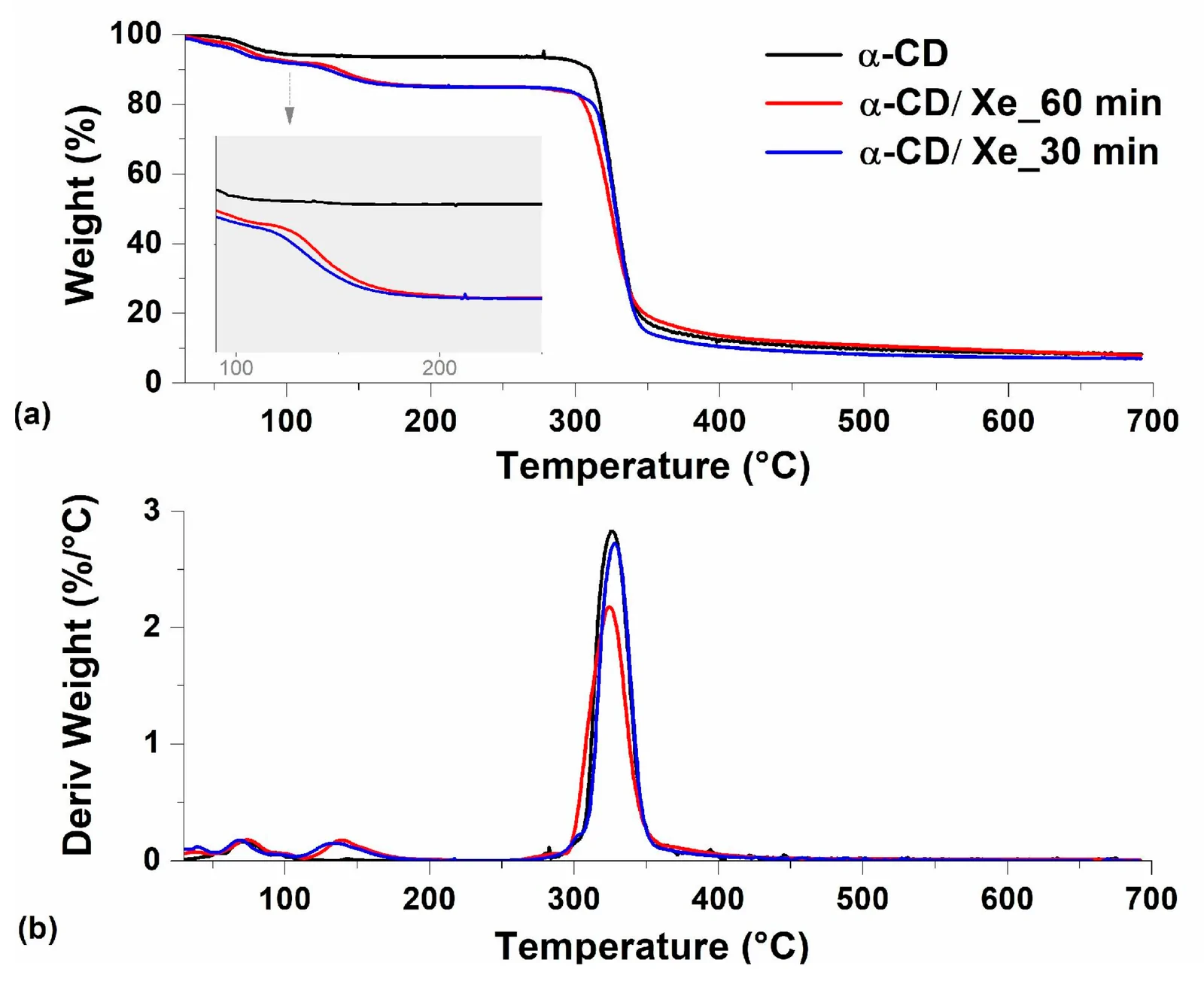
Panel (**a**) shows TGA curves of α-CD and α-CD/Xe obtained at 30 and 60 min. Panel (**b**) shows DTG curves of α-CD and α-CD/Xe obtained at 30 and 60 min.

**Figure 8:**
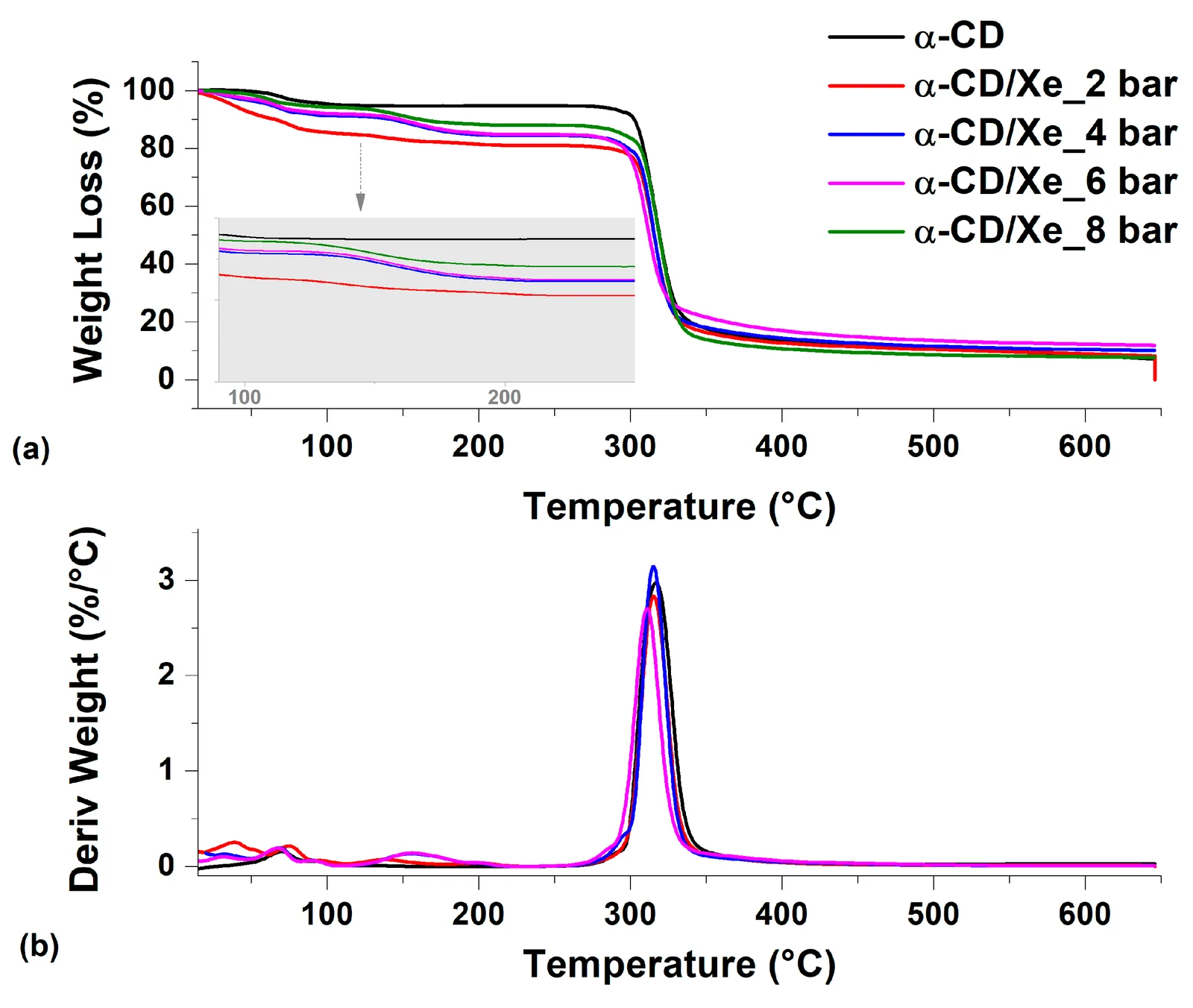
Panel (**a**) shows TGA curves of α-CD and α-CD/Xe obtained at 2, 4, 6 and 8 bar. Panel (**b**) shows DTG curves of α-CD and α-CD/Xe obtained at 2, 4, 6 and 8 bar.

**Figure 9:**
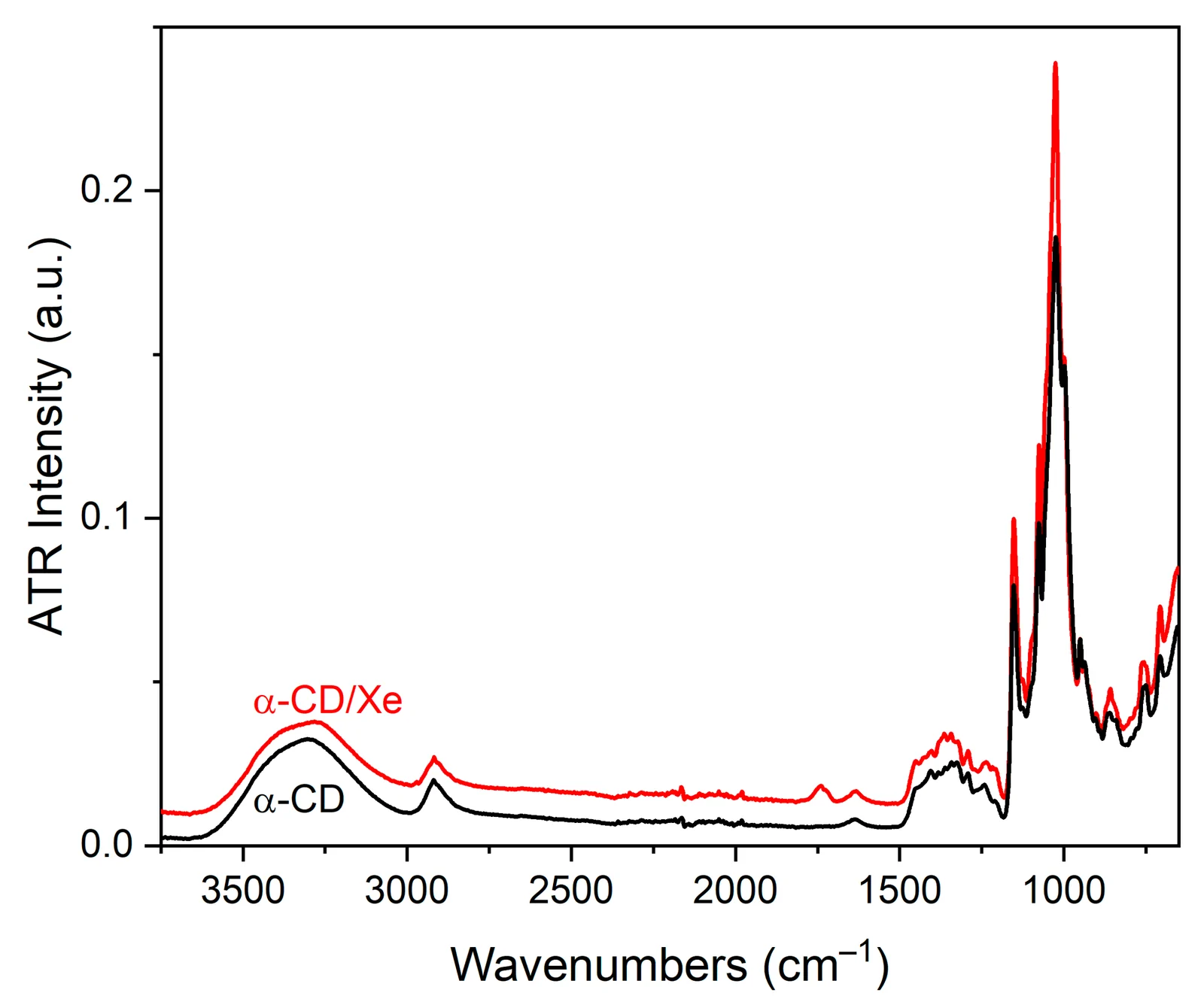
ATR spectra of α-CD and of α-CD/Xe prepared at a Xe loading pressure of 4 bar.

**Figure 10:**
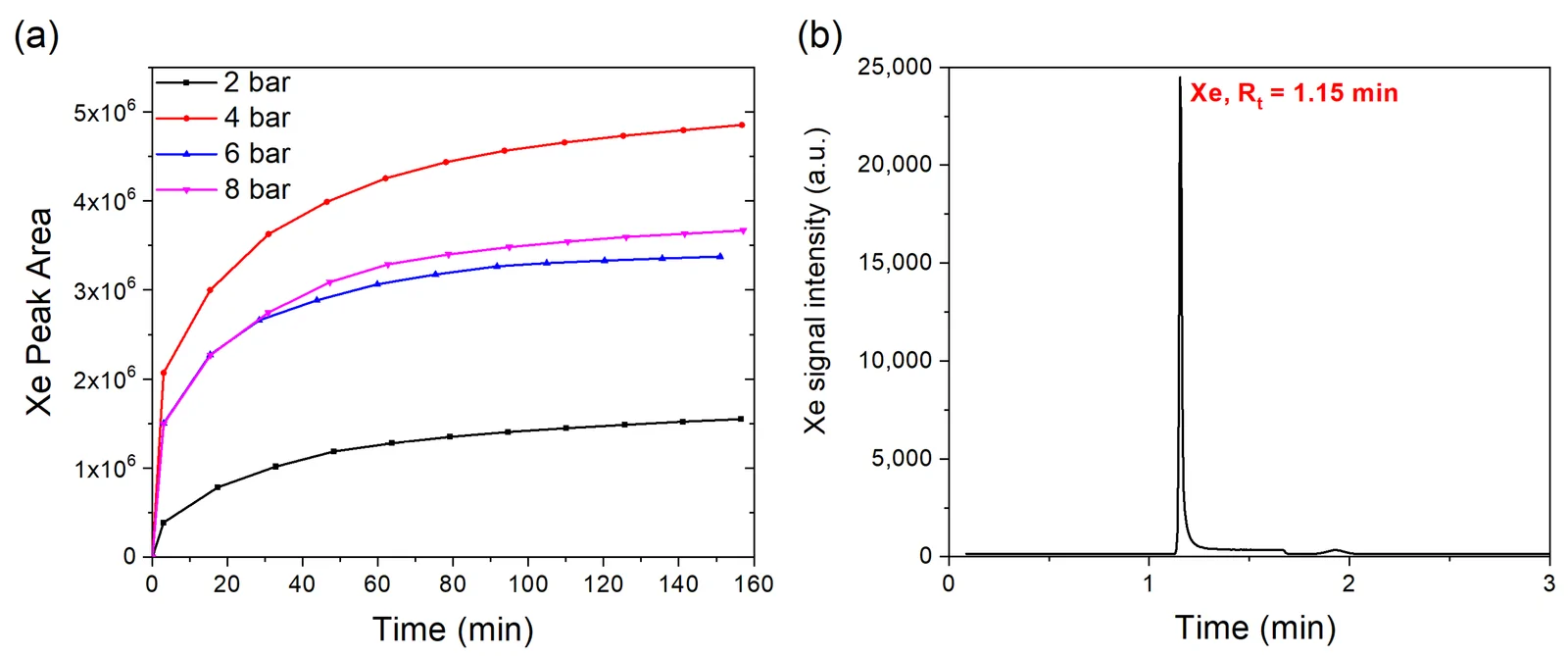
(**a**) Cumulative Xe release, monitored by SPME-GC-MS, from α-CD/Xe complexes prepared by exposing α-CD to Xe at 2, 4, 6, and 8 bar. (**b**) Representative Xe chromatogram obtained for the α-CD/Xe complex prepared at 4 bar.

**Table 1:** Weight loss of the different samples.

Samples		Weight Loss (%)	
	First T ≤ 100 °C	Second 100 °C ≤ T ≤ 200 °C	Third 250 °C ≤ T ≤ 450 °C
α-CD	5.93	-	85.28
α-CD/Xe_30 min	7.52	7.02	75.05
α-CD/Xe_60 min	7.35	7.46	76.99

**Table 2:** TGA Weight loss of the different samples.

Samples		Weight Loss (%)	
	First T ≤ 100 °C	Second 100 °C ≤ T ≤ 200 °C	Third 250 °C ≤ T ≤ 450 °C
α-CD	5.93	-	85.28
α-CD/Xe_2 bar	14.48	4.46	72.66
α-CD/Xe_4 bar	8.72	6.75	74.21
α-CD/Xe_6 bar	8.08	7.02	72.90
α-CD/Xe_8 bar	5.64	6.23	80.39

## Data Availability

The data of this article will be made available by the authors on request.

## Supplementary Materials

The following supporting information can be downloaded at https://www.mdpi.com/article/10.3390/molecules31193554/s1. Figure S1: Initial *non-optimized* geometries studied for the interactions between one, two, and three Xe atoms and α-CD. Figure S2: Initial *non-optimized* geometries studied for the interactions between one, two, and three Xe atoms and α-CD. Figure S3: Initial, *non-optimized* geometries studied in water for the interactions between one, two, and three Xe atoms and α-CD. Figure S4: Initial, *non-optimized* geometries studied in water for the interactions between ten and thirty Xe atoms and α-CD. Figure S5: Initial optimized geometries studied in water for the interactions between one, two, and three Xe atoms and α-CD. Figure S6: Initial optimized geometries studied in water for the interactions between ten and thirty Xe atoms and α-CD. Figure S7: Panel (a): Distance between two xenon atoms from the α-CD c.o.m. calculated during MD simulation in water lasting 2 ns as a function of time, considering the initial α-CD/Xe ratio of 1:3. Panels (b,c): Optimized α-CD/Xe inclusion complexes with a 1:2 stoichiometry formed in water during two different MD runs starting from α-CD:Xe ratios of 1:2 and 1:3, as reported in panels (b) and (c) in Figure S5. The color code is the same in Figure S3. Figure S8: Optimized geometries sampled during the MD simulation in water starting from α-CD and Xe atom ratios of 1:10 and 1:30. Water molecules were omitted for clarity. Table S1: Solvent-accessible surface area values (Å^2^) calculated for some optimized geometries saved during MD simulations performed with different ratios of α-CD to Xe atoms in water. Figure S9: Graphical representation of the solvent-accessible surface area (SASA), calculated in Å^2^, for some of the optimized geometries listed in Table S1; these were saved during molecular dynamics (MD) simulations and performed and optimized using various ratios of α-CD to Xe atoms in water. The SASA is colored according to atom type: carbon in gray, hydrogen in white, oxygen in red, and Xe atoms in petrol blue. Figure S10: Panels (a,b): Plots showing the RDF of 10 and 30 Xe atoms (dark and red lines and symbols) and the RDF of oxygen atoms of water molecule (blue and green lines and symbols) as functions of distance r (in Å) from the center of mass (c.o.m.) of α-CD, calculated during the molecular dynamics (MD) simulations in water starting with α-CD:Xe ratios of 1:10 and 1:30, respectively. Panel (c): Plot showing the RDF of water molecule oxygen and hydrogen atoms (black and red lines and symbols, respectively), calculated during a 2 ns MD simulation in a cell containing one Xe atom and 1200 water molecules, as a function of distance *r* (in Å). Panel (d): Plot showing the RDF of the same water molecule oxygen atom presented in panel (c), compared with the RDF calculated for water molecule oxygen atoms and 30 Xe atoms during the 2 ns MD simulation with an α-CD:Xe ratio of 1:30. Figure S11: Solvent-accessible surface area values (Å^2^), referring exclusively to all α-CD atoms, calculated for selected geometries saved during molecular dynamics (MD) simulations performed in water with various α-CD:Xe atom ratios in water. Specifically, α-CD/Xe inclusion complexes with 1:1, 1:2, 1:3, and 1:4 stoichiometries are reported. Figure S12: pictures of the α-CD solution exposed to Xe at 6 bar and 25 °C at: (a) t = 0 min, (b) t = 2 min, and (c) t = 10 min; (d) resulting powder after lyophilization.

## Abbreviations

The following abbreviations are used in this manuscript:

Xe	Xenon
MM	Molecular Mechanics
MD	Molecular Dynamics
NVT	Number of particles, Volume and Temperature are constant
PBCs	Periodic Boundary Conditions
c.o.m.	center of mass
RDF	Radial Distribution Function
CD	Cyclodextrin
IC	Inclusion complex
H-bond	Hydrogen bond
SASA	Solvent-Accessible Surface Area
AD	Alzheimer’s disease
