# Supplementary material for: Inclusion Complexes Between α-Cyclodextrins and Xenon: Molecular Dynamics Simulations and Experimental Study
Source: Molecules. 2026 Oct 6;31(19):3554. doi: 10.3390/molecules31193554 (PMC13667012; doi:10.3390/molecules31193554)
Supplement: Supplementary file 1 [file molecules-31-03554-s001.zip › molecules-4534138-supplementary.pdf]

Inclusion Complexes between α-Cyclodextrins and Xenon: Molecular Dynamics Simulations and Experimental Study

Francesco Trotta [1], Eya Ben Khalifa [1], Fabrizio Caldera [1], Adrián Matencio [1,], Gjylije Hoti [1], Federico Cesano [1,*] and Giuseppina Raffaini [2,*]

[1] Department of Chemistry, University of Torino, Via P. Giuria 9, 10125 Torino, Italy
[2] Department of Chemistry, Materials and Chemical Engineering "Giulio Natta", Politecnico di Milano, Piazza Leonardo da Vinci 32, 20133 Milano, Italy
* Correspondence: federico.cesano@unito.it; giuseppina.raffaini@polimi.it

Table of Contents

2.1.1. Theoretical study of α-CD/Xe interactions *in vacuo*

Figure S1. Side view in stick representation of the initial *non-optimized* geometries studied for the interactions between one, two, and three Xe atoms and α-CD in panels a), b), and c) respectively. Color codes: C atoms are grey, O atoms are red, Xe atoms are petrol blue, and H atoms are white. S3

Figure S2. Side view in stick representation of the initial *non-optimized* geometries studied for the interactions between one, two, and three Xe atoms and α-CD in panels a) and b) respectively. Color code is the same in Figure S1. S3

2.1.2. Theoretical study of α-CD/Xe interactions in aqueous solution

Figure S3. Side view of the initial, *non-optimized* geometries studied in water for the interactions between one, two, and three Xe atoms and α-CD in panels a), b), and c) respectively. Color codes: all α-CD atoms are in green, O atoms are red, are petrol blue, and H atoms are white. Water molecules and α-CD are represented as stick figures, and Xe atoms are represented as CPK. S4

Figure S4. Side view of the initial, *non-optimized* geometries studied in water for the interactions between ten and thirty Xe atoms and α-CD in panels a), and b) respectively. Color code is the same of Figure S3. S4

Figure S5. Side view of the initial optimized geometries studied in water for the interactions between one, two, and three Xe atoms and α-CD in panels a), b), and c) respectively. Color codes: all α-CD atoms are in yellow, O atoms are red, are petrol blue, and H atoms are white. Water molecules and α-CD are represented as stick figures, and Xe atoms are represented as CPK. Panels d), e), and f) show the potential energy, and Coulomb energy calculated during an MD run lasting for 2 ns in water starting from the geometry in Panels a), b), and c) of Figure S5. S5

Figure S6. Side view of the initial optimized geometries studied in water for the interactions between ten and thirty Xe atoms and -CD in panels a), and b) respectively. Color code is the same of Figure S3. Panels c), and d) show the potential energy, and Coulomb energy calculated during an MD run lasting for 2 ns in water starting from the geometry in Panels a), and b) of Figure S6. S5

Figure S7. Panel a): Distance between two xenon atoms from the α-CD c.o.m. calculated during MD simulation in water lasting 2 ns as a function of time, considering the initial α-CD/Xe ratio of 1:3. Panels (b,c): Optimized α-CD/Xe inclusion complexes with a 1:2 stoichiometry formed in water during two different MD runs starting from α-CD:Xe ratios of 1:2 and 1:3, as reported in panels b) and c) in Figure S5. The color code is the same in Figure S3. S6

Figure S8. Panels a1), b1) and c1) show the side view of the optimized geometries sampled during the MD simulation in water starting from α-CD and Xe atom ratios of 1:10 in stick representation. Water molecules are omitted for clarity. Panels a2), b2) and c2) show the top view in CPK representation, while panels a3), b3) and c3) show the selected distances between Xe atoms in the cavities of α-CD in stick representation. Panel e1) shows the side view of an optimized geometry sampled during the MD simulation in water starting from α-CD and Xe atom ratio of 1:30 ratio in the stick representation. Panels e2) and e3) instead show the same system but viewed from above, from the secondary edge and the primary rim of the α-CD, respectively. S7

Table S1. Solvent-accessible surface area values (Å^2) calculated for some optimized geometries saved during MD simulations performed with different ratios of α-CD to Xe atoms in water. S8

Figure S9. Graphical representation of the solvent-accessible surface area (SASA), calculated in Å^2, for some of the optimized geometries listed in Table S1; these were saved during molecular dynamics (MD) simulations and performed and optimized using various ratios of α-CD to Xe atoms in water. The SASA is colored according to atom type: carbon in gray, hydrogen in white, oxygen in red, and Xe atoms in petrol blue.. S9

Figure S10. Panels (a,b): Plots showing the RDF of 10 and 30 Xe atoms (dark and red lines and symbols) and the RDF of oxygen atoms of water molecule (blue and green lines and symbols) as functions of distance r (in Å) from the center of mass (c.o.m.) of α-CD, calculated during the molecular dynamics (MD) simulations in water starting with α-CD:Xe ratios of 1:10 and 1:30, respectively. Panel (c): Plot showing the RDF of water molecule oxygen and hydrogen atoms (black and red lines and symbols, respectively), calculated during a 2 ns MD simulation in a cell containing one Xe atom and 1200 water molecules, as a function of distance *r* (in Å). Panel (d): Plot showing the RDF of the same water molecule oxygen atom presented in panel (c), compared with the RDF calculated for water molecule oxygen atoms and 30 Xe atoms during the 2 ns MD simulation with an α-CD:Xe ratio of 1:30. **S10**

Figure S11. Solvent-accessible surface area values (Å^2), referring exclusively to all α-CD atoms, calculated for selected geometries saved during molecular dynamics (MD) simulations performed in water with various α-CD to Xe atom ratios in water. Specifically, α-CD/Xe inclusion complexes with 1:1, 1:2, 1:3, and 1:4 stoichiometries are reported. S11

Figure S12. Pictures of the α-CD solution exposed to Xe at 6 bar and 25 °C at: (a) t = 0 min, (b) t = 2 min, and (c) t = 10 min; (d) resulting powder after lyophilization. S12

2.1.1. Theoretical study of α-CD/Xe interactions *in vacuo*

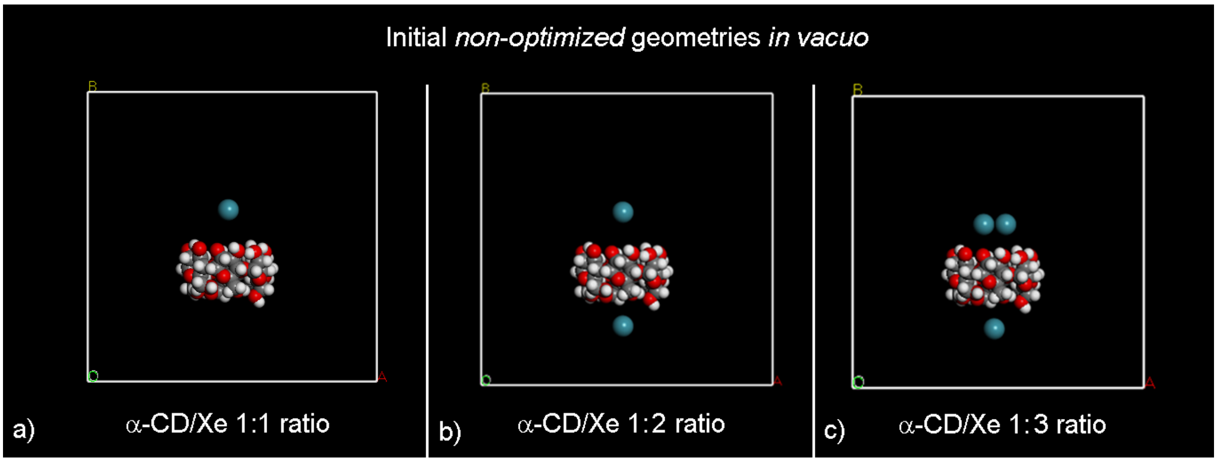

Figure S1. Side view in stick representation of the initial *non-optimized* geometries studied for the interactions between one, two, and three Xe atoms and α-CD in panels a), b), and c) respectively. Color codes: C atoms are grey, O atoms are red, Xe atoms are petrol blue, and H atoms are white.

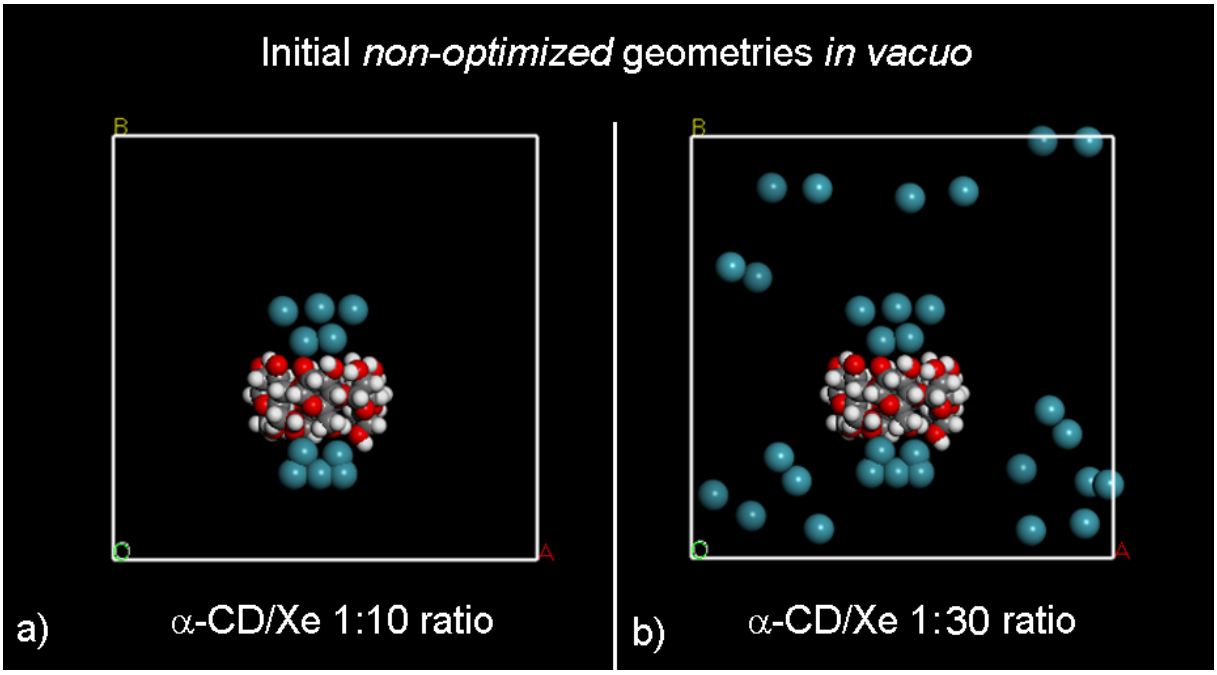

Figure S2. Side view in stick representation of the initial *non-optimized* geometries studied for the interactions between one, two, and three Xe atoms and α-CD in panels a) and b) respectively. Color code is the same in Figure S1.

2.1.2. Theoretical study of α-CD/Xe interactions in aqueous solution

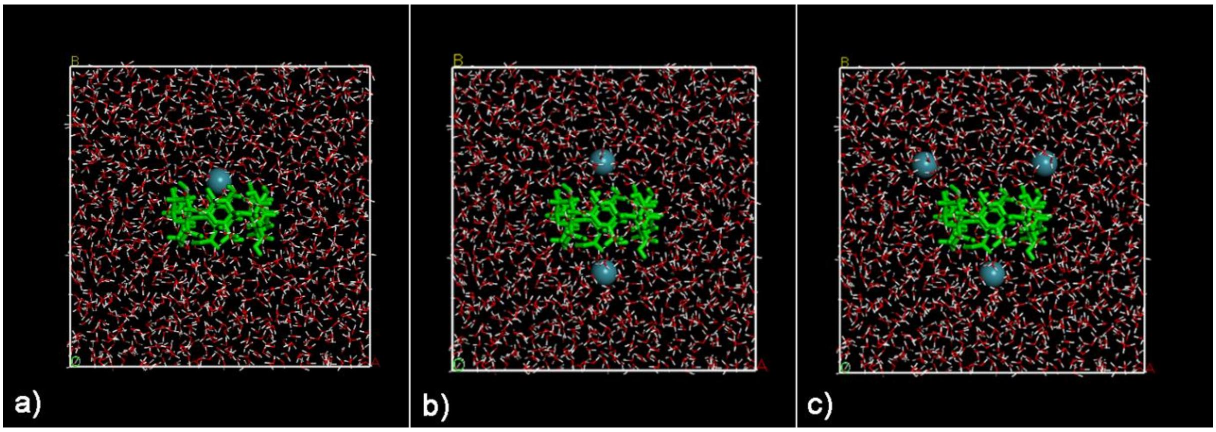

Figure S3. Side view of the initial, *non-optimized* geometries studied in water for the interactions between one, two, and three Xe atoms and α-CD in panels a), b), and c) respectively. Color codes: all α-CD atoms are in green, O atoms are red, are petrol blue, and H atoms are white. Water molecules and α-CD are represented as stick figures, and Xe atoms are represented as CPK.

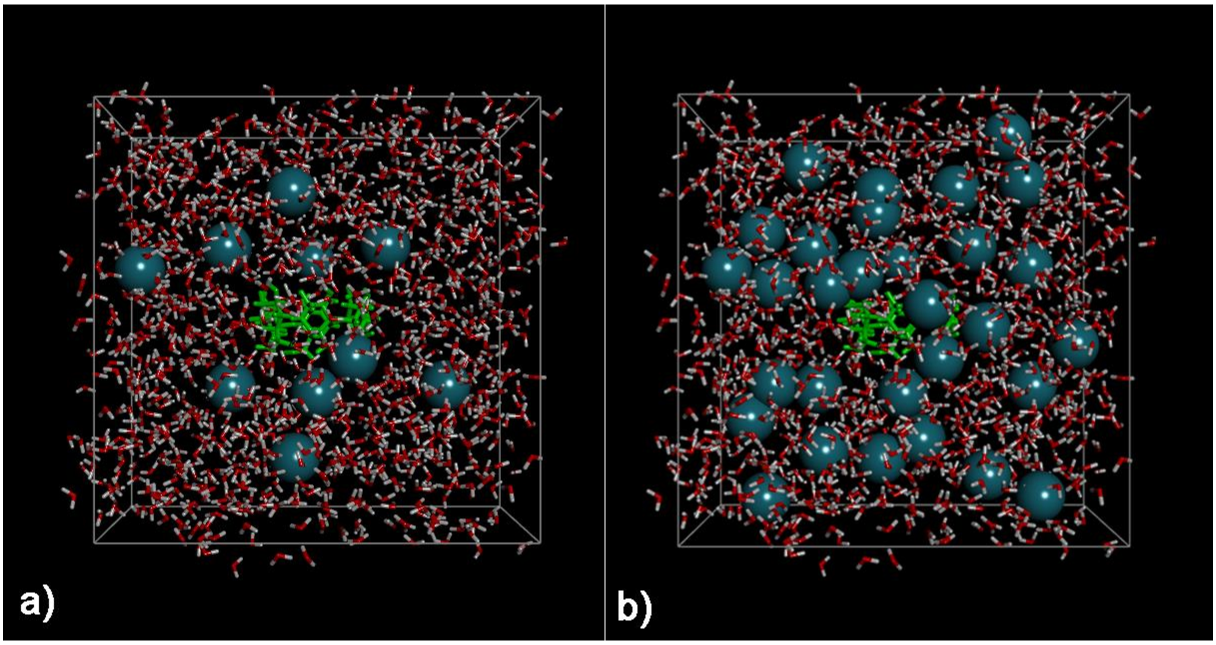

Figure S4. Side view of the initial, *non-optimized* geometries studied in water for the interactions between ten and thirty Xe atoms and α-CD in panels a), and b) respectively. Color code is the same of Figure S3.

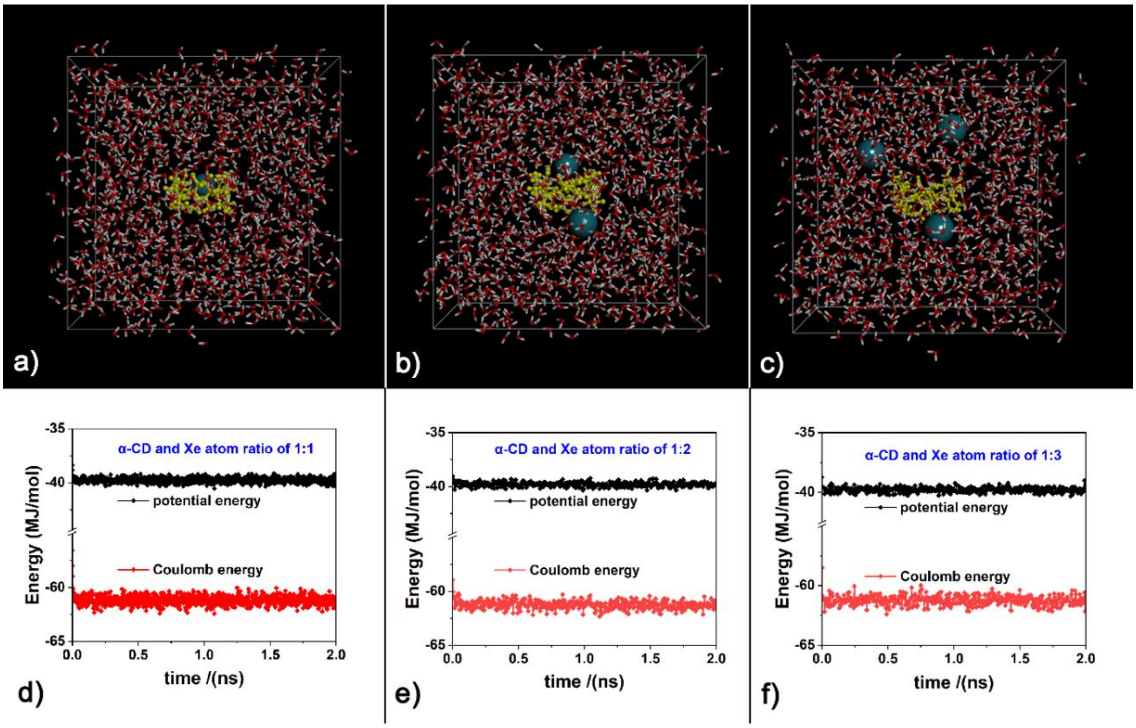

Figure S5. Side view of the initial optimized geometries studied in water for the interactions between one, two, and three Xe atoms and α-CD in panels a), b), and c) respectively. Color codes: all α-CD atoms are in yellow, O atoms are red, are petrol blue, and H atoms are white. Water molecules and α-CD are represented as stick figures, and Xe atoms are represented as CPK. Panels d), e), and f) show the potential energy, and Coulomb energy calculated during an MD run lasting for 2 ns in water starting from the geometry in Panels a), b), and c) of Figure S5.

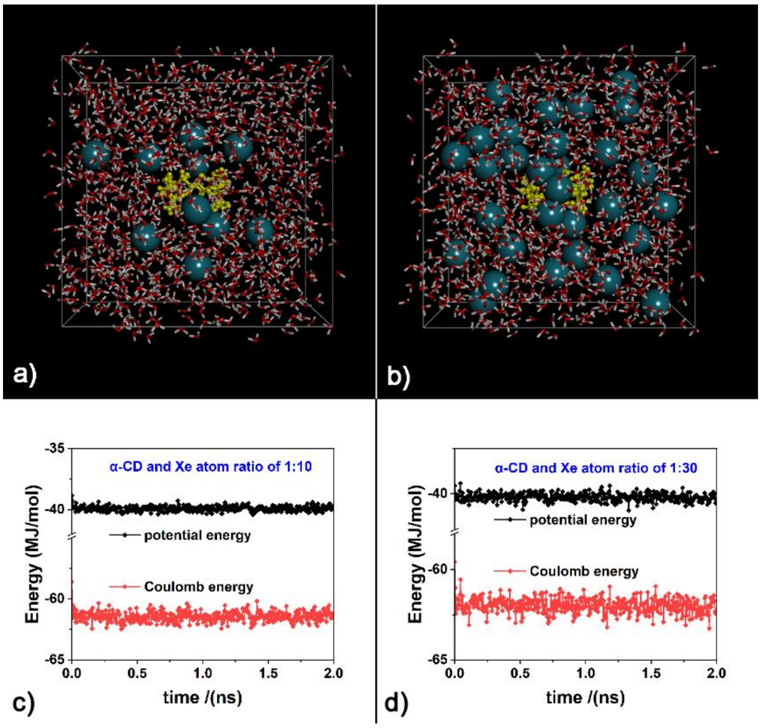

Figure S6. Side view of the initial optimized geometries studied in water for the interactions between ten and thirty Xe atoms and α-CD in panels a), and b) respectively. Color code is the same of Figure S3. Panels c), and d) show the potential energy, and Coulomb energy calculated during an MD run lasting for 2 ns in water starting from the geometry in Panels a), and b) of Figure S6.

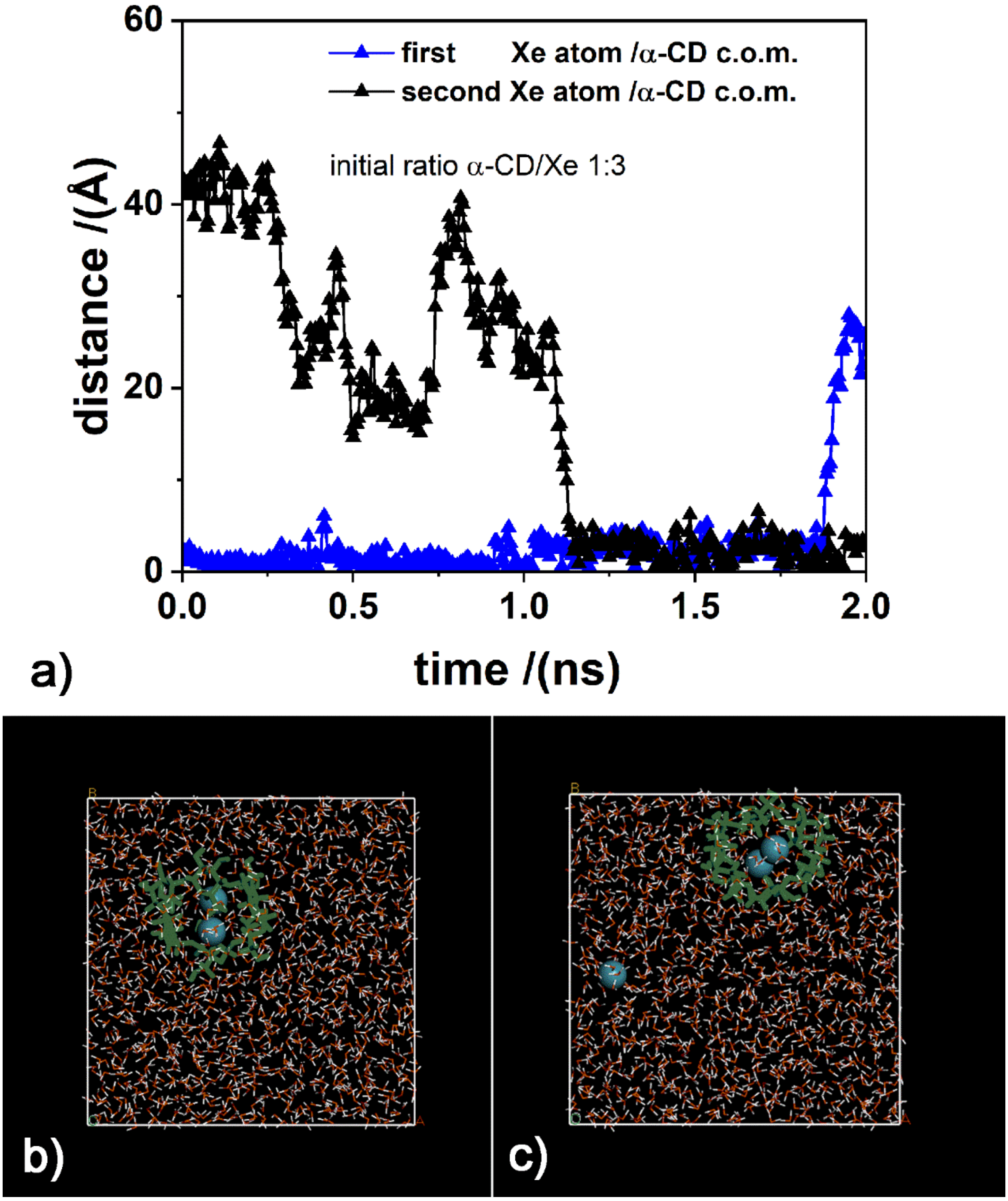

Figure S7. Panel a): Distance between two xenon atoms from the α-CD c.o.m. calculated during MD simulation in water lasting 2 ns as a function of time, considering the initial α-CD/Xe ratio of 1:3. Panels (b,c): Optimized α-CD/Xe inclusion complexes with a 1:2 stoichiometry formed in water during two different MD runs starting from α-CD:Xe ratios of 1:2 and 1:3, as reported in panels b) and c) in Figure S5. The color code is the same in Figure S3.

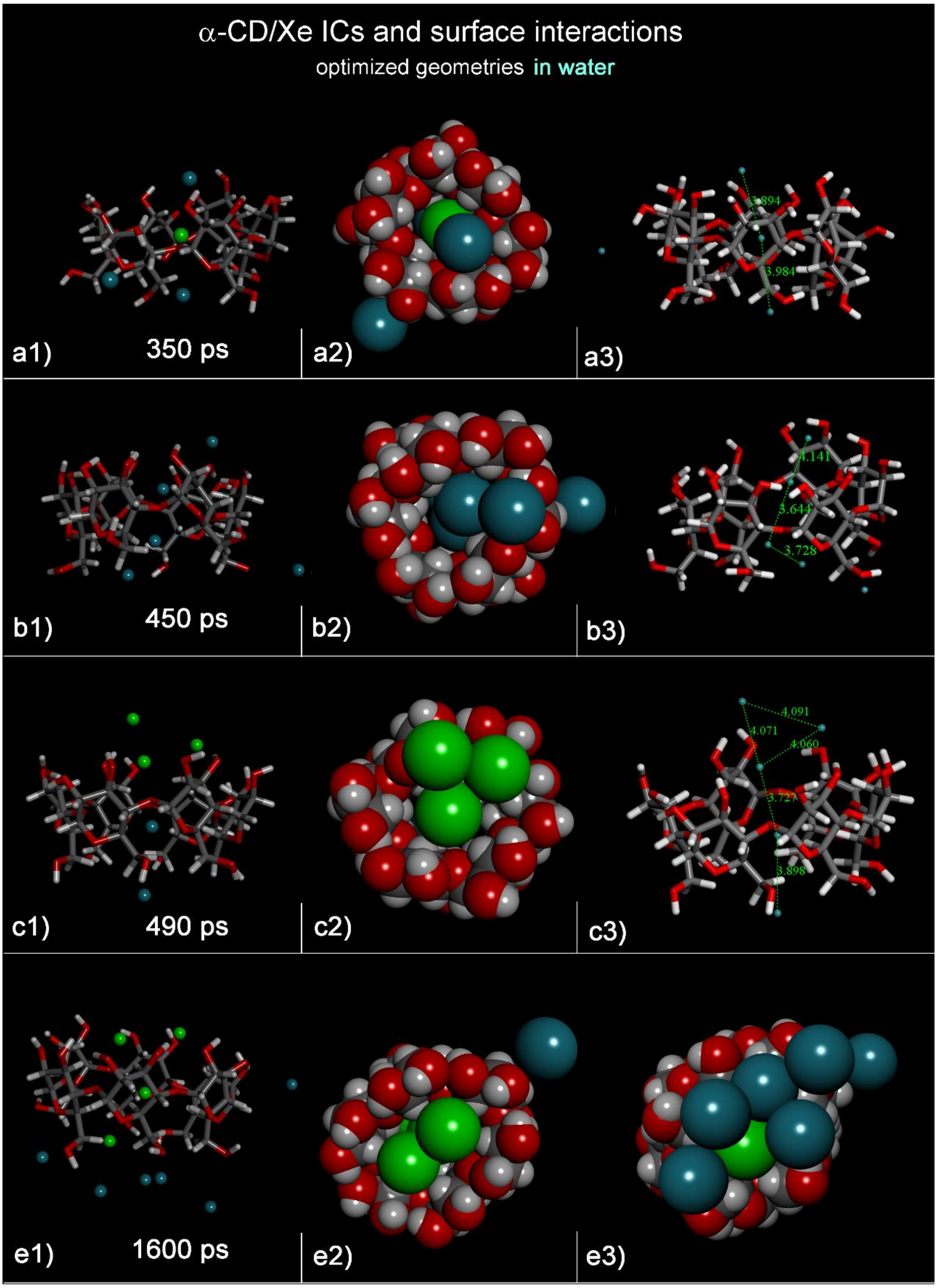

Figure S8. Panels a1), b1) and c1) show the side view of the optimized geometries sampled during the MD simulation in water starting from α-CD to ~~and~~ Xe atom ratio of 1:10 in stick representation. Water molecules are omitted for clarity. Panels a2), b2) and c2) show the top view in CPK representation, while panels a3), b3) and c3) show the selected distances between Xe atoms in the cavities of α-CD in stick representation. Panel e1) shows the side view of an optimized geometry sampled during the MD simulation in water starting from α-CD and Xe atom ratio of 1:30 ratio in the stick representation. Panels e2) and e3) instead show the same system but viewed from above, from the secondary edge and the primary rim of the α-CD, respectively.

Table S1. Solvent-accessible surface area values (Å²) calculated for some optimized geometries saved during MD simulations performed with different ratios of α-CD to Xe atoms in water.

System optimized in water	SASA (Å²)
α-CD	683
α-CD/Xe in 1:1 stoichiometry	643
α-CD/Xe in 1:2 stoichiometry	608<br>Distance between two encapsulated Xe atoms = 3.6 Å
"	611<br>Distance between two encapsulated Xe atoms = 3.7 Å
"	630<br>Distance between two encapsulated Xe atoms = 3.9 Å
"	620<br>Distance between two encapsulated Xe atoms = 4.1 Å
"	635<br>Distance between two encapsulated Xe atoms = 4.3 Å
α-CD/Xe in 1:3 stoichiometry	606
"	603
α-CD interacting with 4 Xe atoms	583
"	590
α-CD interacting with 6 Xe atoms	602
α-CD interacting with 9 Xe atoms	543

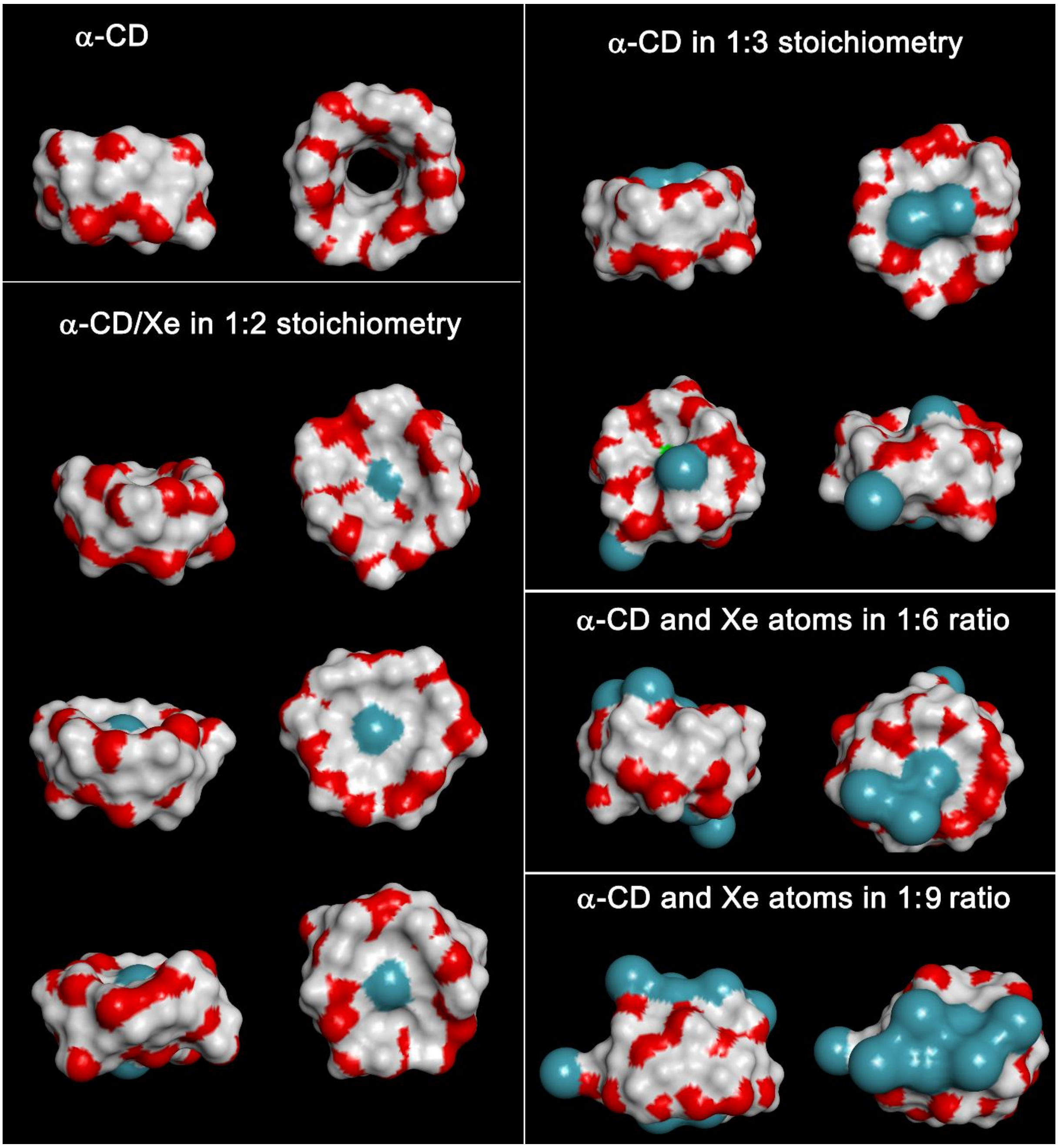

Figure S9. Graphical representation of the solvent-accessible surface area (SASA), calculated in Å², for some of the optimized geometries listed in Table S1; these were saved during molecular dynamics (MD) simulations and performed and optimized using various ratios of α-CD to Xe atoms in water. The SASA is colored according to atom type: carbon in gray, hydrogen in white, oxygen in red, and Xe atoms in petrol blue.

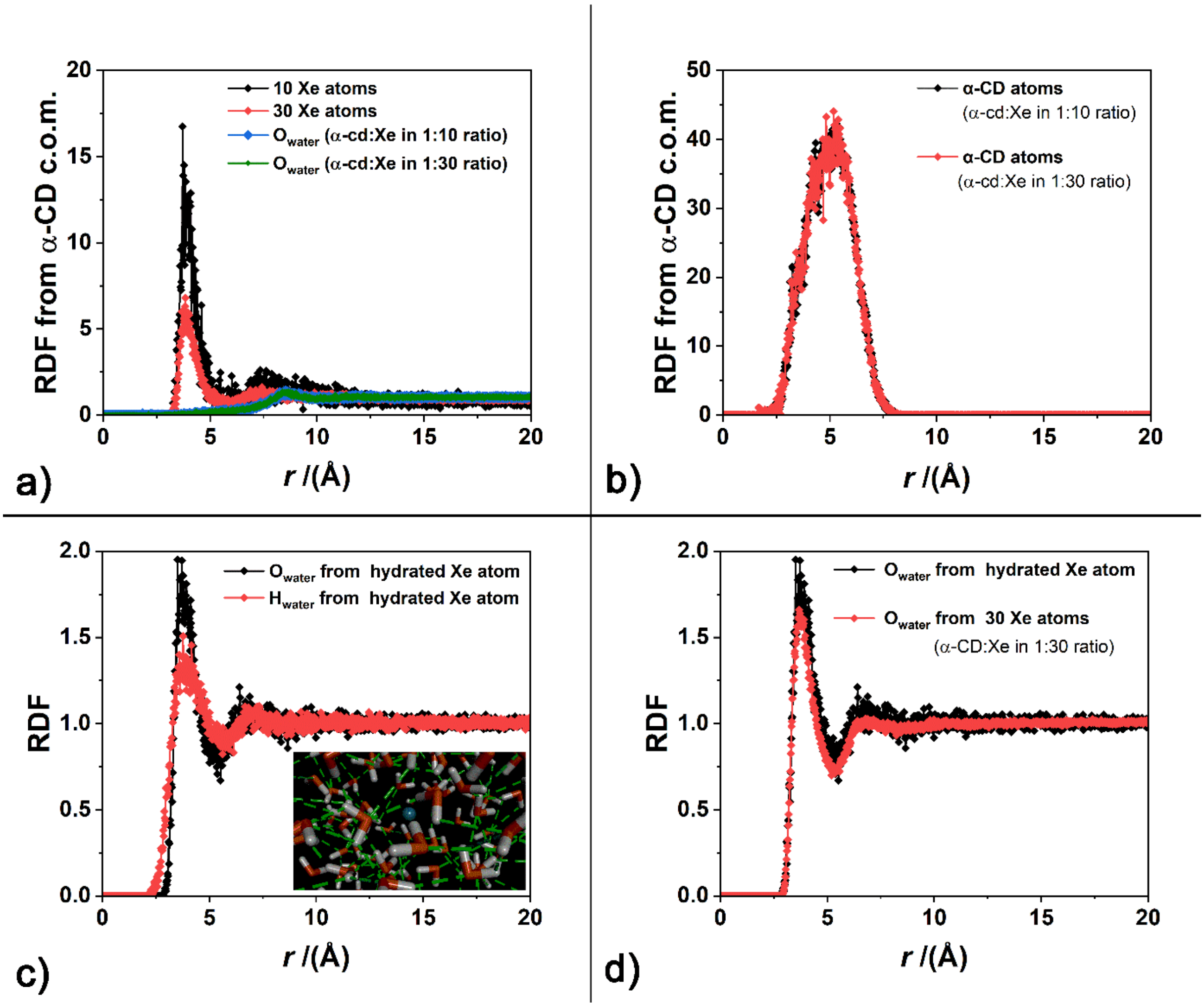

Figure S10. Panels (a,b): Plots showing the RDF of 10 and 30 Xe atoms (dark and red lines and symbols) and the RDF of oxygen atoms of water molecule (blue and green lines and symbols) as functions of distance r (in Å) from the center of mass (c.o.m.) of α-CD, calculated during the molecular dynamics (MD) simulations in water starting with α-CD:Xe ratios of 1:10 and 1:30, respectively. Panel (c): Plot showing the RDF of water molecule oxygen and hydrogen atoms (black and red lines and symbols, respectively), calculated during a 2 ns MD simulation in a cell containing one Xe atom and 1200 water molecules, as a function of distance r (in Å). Panel (d): Plot showing the RDF of the same water molecule oxygen atom presented in panel (c), compared with the RDF calculated for water molecule oxygen atoms and 30 Xe atoms during the 2 ns MD simulation with an α-CD:Xe ratio of 1:30.

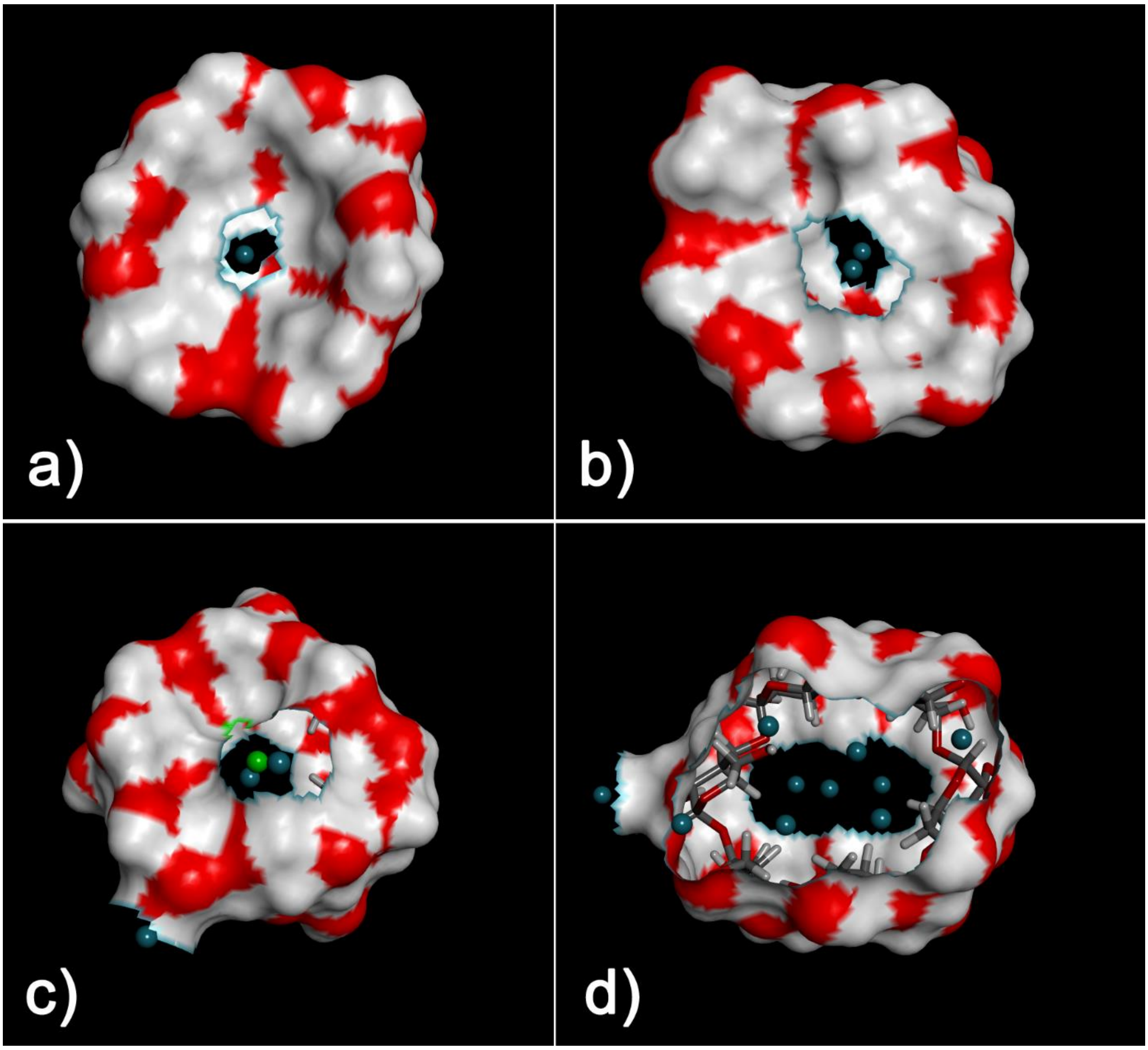

Figure S11. Solvent-accessible surface area values (Å^2), referring exclusively to all α-CD atoms, calculated for selected geometries saved during molecular dynamics (MD) simulations performed in water with various α-CD to Xe atom ratios in water. Specifically, α-CD/Xe inclusion complexes with 1:1, 1:2, 1:3, and 1:4 stoichiometries are reported.

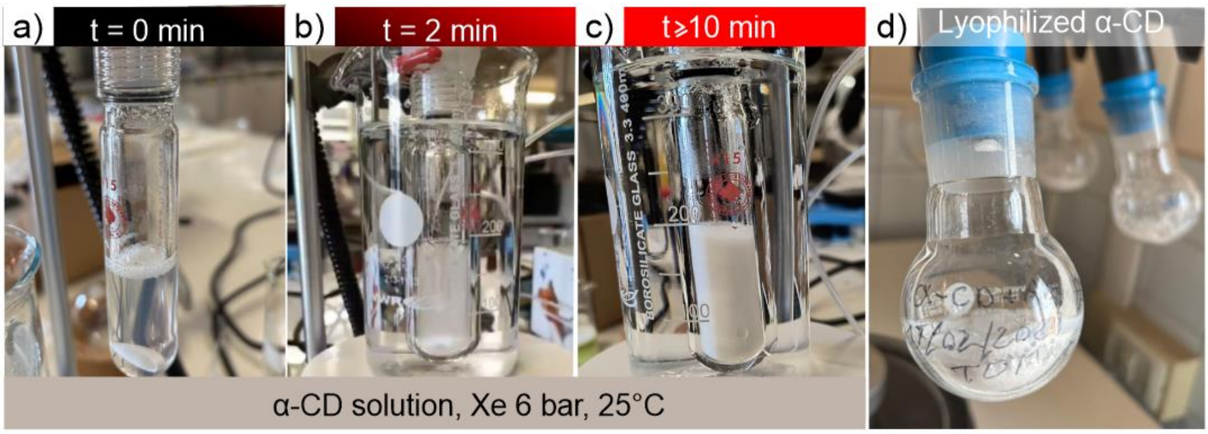

Figure S12. Pictures of the α-CD solution exposed to Xe at 6 bar and 25 °C at: (a) t = 0 min, (b) t = 2 min, and (c) t = 10 min; (d) resulting powder after lyophilization.
